# Land and sea transport options for the installation of green artificial reefs (GARs) in shallow waters: a Galician case study

**DOI:** 10.1038/s41598-024-53183-0

**Published:** 2024-02-01

**Authors:** Juan José Cartelle Barros, Alicia Munín-Doce, Laura Castro-Santos, Javier Lamas, Luis Carral

**Affiliations:** 1 Departamento de Ciencias da Navegación e Enxeñaría Mariña, Escola Politécnica de Enxeñaría de Ferrol, Universidade da Coruña, Campus Industrial de Ferrol, CITENI, Esteiro, 15471 A Coruña, Spain; 2 Departamento de Enxeñaría Naval e Industrial, Escola Politécnica de Enxeñaría de Ferrol, Universidade da Coruña, Campus Industrial de Ferrol, CITENI, Esteiro, 15471 Ferrol, Spain; 3 Anta Norte, Lugar Avenida Mestre Manuel Gómez Lorenzo, 30, Vedra, 15885 A Coruña, Spain

**Keywords:** Climate-change impacts, Ecosystem services, Engineering

## Abstract

The aim of the present paper is to propose a new methodology for the production and installation of green artificial reefs (GARs) in shallow waters, with special attention to the transport stages. The process includes both onshore (manufacturing, road transport and unload at port) and offshore (load at port, sea transport, positioning, and deployment tasks) stages. Two different types of truck were analysed for the road transport. Furthermore, three different options were considered for sea transport: a workboat powered by liquefied natural gas, a barge using diesel (0.1% sulphur) as fuel, and an electric specific design barge. A simulation tool called AGARDO (Automatic Green Artificial Reef Deploy Optimisation) was developed for such a purpose. An estuary located in Galicia (North-West of Spain), where 180 GAR units must be installed, has been considered as case study. AGARDO was used to obtain results concerning process total time, equivalent CO_2_ emissions and costs for different scenarios. Consequently, the use of the proposed methodology allows the decision-maker to select the best option in terms of costs, emissions and time. AGARDO can be easily adapted to other case studies, with different onshore and offshore options.

## Introduction

Marine ecosystems around the world have undergone considerable degradation due to several factors such as climate change, unsustainable fishing patterns, or illegal trawling^1–3^, among others. Consequently, some natural resources are being gradually depleted^4^. In this sense, Artificial Reefs (ARs) emerge as promising options for: protecting and restoring habitats, enhancing biodiversity, increasing marine biomass and fish populations, reducing erosion, managing water quality, providing sustainable fishing opportunities and also promoting tourism and recreational activities (recreational fishing, surfing, diving or boating)^4–14^.

ARs can be defined as manmade structures placed on the seabed with the aim of imitating the most relevant characteristics of natural reefs^12–15^. At a more detailed level, it is possible to differentiate between Opportunity Artificial Reefs (OARs) and Conventional Artificial Reefs (CARs). The former are artificial reefs that were not specifically designed and constructed for restoring marine ecosystems. Its use is based on the principle that any submerged structure can, to a certain extent, act as a natural reef^16^. Consequently, OARs can be intentionally sunk out-of-service vessels, structures made of used tires^17^, or even offshore installations from the oil and gas industry^15^, among other options. On the other hand, CARs are specifically designed for improving living marine resources. They are usually blocks of different geometries and sizes with several holes for both improving nutrient circulation and providing shelter. CARs are usually made of concrete, although there are other alternatives^10^. For some time now, the possibility of incorporating waste (mussel or oyster shells, among others) and recycled materials (fiberglass wool, rock wool, shot or eucalyptus fibres) as partial substitutes of conventional materials (sand, cement and steel) has been studied^16–18^. In this way, CARs become green artificial reefs (GARs), which fall within the framework of sustainable development and circular economy, with positive environmental and economic implications^14^.

A considerable number of marine ecosystems enhance programs have been developed in different countries such as France^19,20^, Italy^6^, Japan^7^, Portugal^21,22^, South Korea^5,23^, Spain^1^, Taiwan^24^, or United States of America^15,25^, among others.

Galician coasts (North-West of Spain) stand out for their geographical characteristics and for being an important source of resources and biological diversity^16,26^. The term “ría” is usually used to refer to Galician estuaries^26^. In this line, Galicia is a heavily dependent region on the fishing and shellfish industries^16,27^. In fact, Galicia is the world’s second largest mussel producer^16^ and it is, to a large extent, responsible for Spain’s third place in the international fishing market^16,28^. Nevertheless, Galician rías have experienced a degradation of their marine ecosystems, which has generated negative socio-economic impacts on the region. By way of example, in the particular case of artisanal fishing, official statistics show that in recent years there has been a steady decline in the volume of catches of certain species, causing massive losses year after year^16^. Consequently, the implementation of an artificial reef program for enhancing Galician estuaries is of paramount importance.

The reader should bear in mind that manufacturing, transporting, and installing a great number of ARs (such as that needed for Galicia) requires substantial investment and time. Furthermore, it can also generate considerable negative impacts on the environment because of raw material and energy consumption as well as pollutant gas emissions. Therefore, in this type of action, it is necessary to analyse in detail times, emissions, and costs. By doing so, the positive impacts of the enhance program will not be overtaken by the potential negative ones.

On the other hand, in the specialised literature, a great number of authors have performed studies on ARs, adopting different approaches and pursuing a variety of objectives as can be deducted from Lee et al.^29^. Many studies asses the performance of ARs by developing experimental or field observations, years after their installation. As an example, Polovina and Sakai^30^ analysed the capacity of ARs to increase fishery production in the island of Hokkaido (Japan). The authors considered the case of flatfish and octopus. The reader can find more examples in Bulleri et al.^31^ or in Fariñas-Franco et al.^32^, among many others^29^. There are also works with a similar aim, based on modelling rather than field studies. For instance, the one carried out by Campbell et al.^33^, in which the authors created a model to simulate how ARs affect the population dynamics of several fish species.

Other research studies focus on ARs’ materials. Chen et al.^34^ analysed the mechanical properties, workability and affinity of a new concrete for ARs. Similarly, Carral et al.^16^ proposed the use of waste materials from industrial processes associated with the maritime sector to replace concrete aggregates. The authors demonstrated the suitability of their proposal by mechanical testing and a statistical analysis. In a similar vein is the study developed by Carral et al.^17^. In this case, the authors created a model based on a multi-criteria decision-making method to assess the sustainability index of different GARs (Green Artificial Reefs).

Considerable efforts have also been made to study flow fields around ARs, their stability or related issues. Duzbastilar and Utku^9^ analysed the stability of cube and pipe AR designs by using wave data from the Aegean Sea, the Black Sea and the Mediterranean Sea. Kim et al.^35^ investigated the structural responses and wake regions of 24 different AR types. The drag coefficients of the same 24 designs were studied by Woo et al.^8^. A finite volume flow analysis was performed for such a purpose. Some authors also estimated the wake volumes of different AR designs^10,36^.

There are also studies addressing placement and installation processes of ARs. By way of example, Kim et al.^13^ proposed 6 different placement models for AR sets. Nevertheless, the authors focused on the characterisation of wake and upwelling regions. Lan et al.^4^ created a new mathematical model to design the spatial configuration of ARs. However, the aim of the proposed algorithm is to maximise the fractal dimension. A considerable number of authors analysed the process of installing ARs, but focusing on the settlement suffered by each AR unit^12^. It depends on several factors such as the seabed soil composition, the geometry of the AR, the type of installation (free-fall or guided way), among others. Some of them are short-term studies, addressing the initial settlement^11,23^, while others estimated the burial volume years after the installation^5,37^.

The site selection of ARs was also considered in the existing literature. Carral et al.^38^ proposed a new methodology to identify the best locations for AR groups, by taking into account environmental and socio-economic factors. The authors used a geographic information system (GIS) together with computational algorithms for such a purpose. In a similar line is the study previously developed by Barber et al.^39^. In this case, biological and physical field measurements were combined with exclusion mapping.

On the other hand, to the best of the authors’ knowledge, there are hardly any studies addressing the manufacture, transport and installation processes of ARs in detail, by exploring different alternatives or by taking into account economic, environmental and time issues. One of the few examples is the one performed by Carral et al.^26^. The authors conducted a preliminary study to assess the contribution to climate change derived from a marine ecosystem enhancement program in the estuary region of Galicia. Nevertheless, this study presents some limitations. On the one hand, only two different means of transport were considered for the maritime transport. On the other, costs and times were not included in the study. The reader should note that offshore operations, such as the installation of ARs, are highly dependent on weather windows^40^. Consequently, estimating the total installation time is a key issue, particularly in seas with harsh weather conditions during many months of the year. Furthermore, Carral et al.^26^ did not explained in detail the deployment operations. One may think that they are similar to the ones developed in other offshore industries^15,40,41^. Nevertheless, the main difference is that ARs are usually deployed in shallow waters, while the majority of subsea equipment is for deep waters. Therefore, both the installation methods and their costs may differ considerably.

In view of the above, the main objective of this study is to present a new methodology for analysing the production, transport and installation of GARs in shallow waters by taking into account costs, timescales and equivalent CO_2_ emissions. This methodology has materialised in the form of a simulation tool called AGARDO (Automatic Green Artificial Reef Deploy Optimisation). It includes both the onshore (manufacturing, road transport and unload at port) and the offshore (loading process at port, maritime transport, positioning, pre-deployment and deployment tasks) stages^42^. Two different types of trucks were considered for the road transport: (i) rigid truck with three axles and (ii) an articulated option with 5 axles. For the sea transport, three different alternatives were modelled (i) a workboat powered by liquefied natural gas, (ii) a barge using diesel (0.1% sulphur) as fuel, and (iii) an electric specific design barge), although it is possible to include more options in future developments. The case study of Galician Estuaries was considered, in particular, the installation of 180 GAR units for Ares-Betanzos ría. A simulation process was carried out, analysing 144 different scenarios. AGARDO can be easily applied to other different case studies. Furthermore, it is also simple to include modifications in this tool to model other onshore and offshore options. The remaining of the paper is organised as follows. In Section "Materials and methods: AGARDO simulation tool", the AGARDO tool is presented and described in detail. All the information linked to the case study is included in Section "Case study". The results are presented and discussed in Section "Results and discussions". Finally, the main conclusions as well as the potential future developments are shown in Section "Conclusions and future developments".

## Materials and methods: AGARDO simulation tool

**Figure 1:**
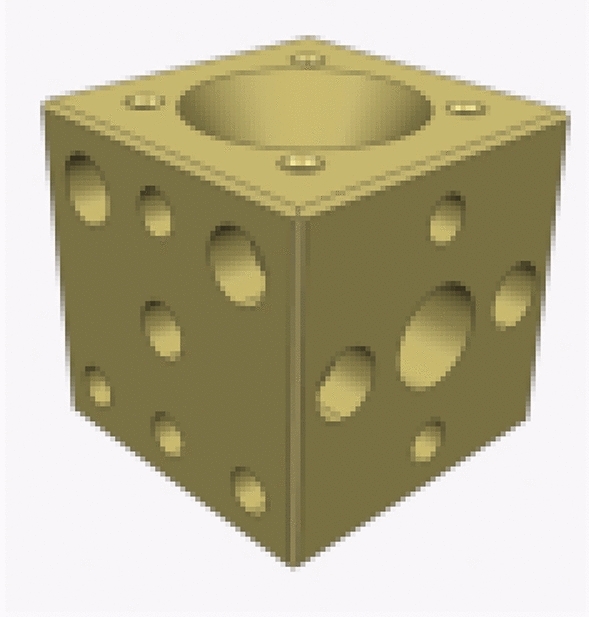
GAR design. Source: own elaboration using SolidWorks software, 2017 SP5 version (https://www.solidworks.com/).

First, it is necessary to provide some general information about the green artificial reef units to be installed, since they will condition, to certain extent, the development of the AGARDO simulation tool. Nevertheless, the reader should bear in mind that AGARDO can be modified to be used with other types of artificial reefs different from the ones considered here. In this sense, the GAR (Green Artificial Reef) unit taken into account in this study has been described in the PROARR research project^26^. It is a concrete cube (edge of 1.5 m) with holes and nest cavities (Fig. 1). Its total mass is about 5 tons and some percentages of its conventional materials (cement, grave, sand or steel frames) were replaced by alternative options such as oyster and mussel shells or eucalyptus vegetable fibres. On the other hand, the GAR units will be installed in groups of 60 modules, configuring a rectangular geometry. The units must be separated from each other by a distance between 5 and 10 m, being acceptable a deviation of up to 1.5 m with respect to its desired theoretical position.

On the other hand, it is also necessary to explain the general process that each GAR unit follows. In this line, it is important to note that the procurement and the transport steps of the materials employed in the manufacture of GARs is out of the scope of this study. Consequently, the corresponding costs, time and emissions are not included in AGARDO.

**Figure 2:**
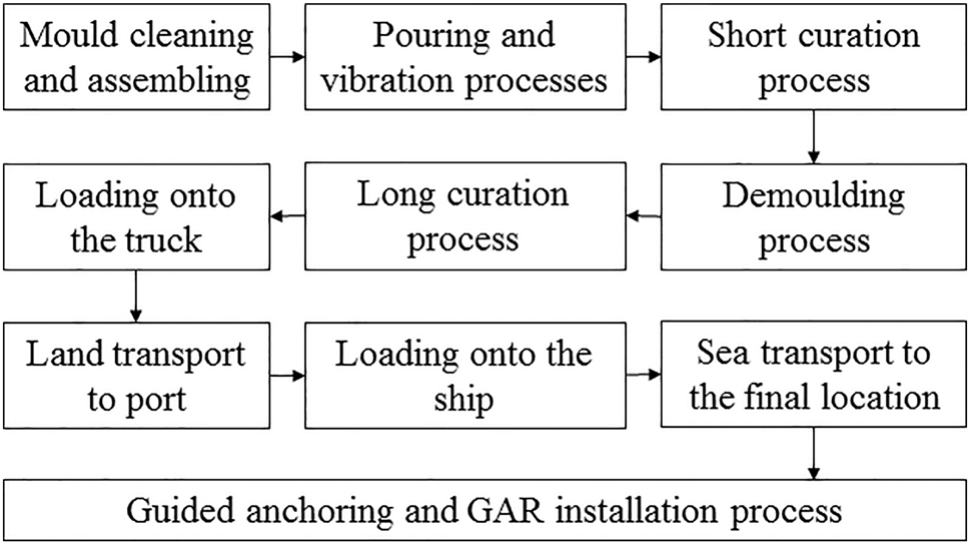
General scheme of the process that each GAR unit experiments. Source: own elaboration.

The production of each GAR unit starts with a process of pouring concrete in a mould specially designed for the geometry shown in Fig. 1. Each mould must be previously assembled and, if necessary, cleaned. Then a vibration process is applied to prevent the occlusion of air bubbles. Next, the mould is transported to an area where the GAR unit undergoes an initial curing process (short curing) with the objective of reaching the necessary hardness for the demoulding step. After that, the mould is removed and the GAR is subsequently transported to the long curation area. When this second curing finishes, the GAR is ready for the ground transport stage. Therefore, it is transported to the loading area. The fully cured units are then loaded onto the trucks and transported to the port. In the next stage, GAR units are loaded onto the ships to be finally transported to the final location for their installation. The reader can find a general scheme of this process in Fig. 2.

AGARDO has been designed in such a way that modifications can be made easily and quickly by anyone. Therefore, it was developed and implemented in a free and open-source software (FOSS) tool. In particular, Python language was used. AGARDO is based on a discrete event simulation technique, so it was necessary to employ the SimPy library^43^. In SimPy all components are modelled with processes that are based on the Python’s generator functions. This library has been successfully used in other existing studies with different purposes^44–46^.

The mathematical model of the process considers the onshore and the offshore stages. A JIT (Just in Time) production mode has been sought. All the parts of the tool work at the same time in order to minimise or even eliminate the need for intermediate warehouses and buffers, especially in the locations of greater logistical impact (deployment ports). However, there can be particular cases in which it is not possible to completely eliminate those intermediate buffers. Consequently, this possibility is also included in AGARDO.

On the other hand, it is possible to say that AGARDO works as a stand-alone tool that models the whole process in an integrated way. Nevertheless, at a conceptual level, three modules can be distinguished: the manufacturing module or Module 1, the land transport module or Module 2, and the sea transport module or Module 3. In the first Section of Appendix A, the reader can find additional information about these modules, as well as their corresponding flowcharts.

On the other hand, the user must define the values that a set of initial parameters adopt. These parameters depend on the case study to be analysed. Some of them can be treated as decision variables with the objective of simulating all those scenarios that are feasible from a practical point of view. After all realistic simulations have been carried out, the user will obtain a set of results for each one of the scenarios. Consequently, it will be possible to select the best option according to different criteria such as minimum cost, time or CO_2-eq._ emissions. Another alternative is to choose a balanced solution, that is, the one that allows the entire process to be completed within the required timeframe (weather windows) and with reasonable costs and emissions. In this respect, the costs as well as the CO_2-eq._ emissions derived from the manufacturing process are not quantified. There are several reasons to justify this: (i) only one single GAR design is considered, (ii) alternative production processes have not been implemented in AGARDO, (iii) the same dosage is always used for each GAR, among others. This could be modified if, for example, one wants to quantify the potential emission mitigation derived from installing solar photovoltaic panels in the manufacturing plant. Table 1 lists the parameters of the AGARDO tool.

**Table 1:** AGARDO set of parameters, including their units of measurement and description.

Parameter	Units	Description
*N*_*GAR*_	GAR units	Total number of GAR units to be installed
*N*_*B*_	GAR units	Additional number of GAR units to be manufactured to replace all those that may break during the most critical phases (demoulding stage, loading and unloading processes, among others). It can also be defined as a percentage of *N*_*GAR*_
*N*_*M*_	moulds	Number of moulds
*P*_*NM*_	–	Percentage of available moulds used in the manufacturing process
*C*_*M*_	€/mould	Production price of each mould
*T*_*P*_	h	Average time associated with the following tasks: assembling and cleaning the mould and concrete pouring
*T*_*V*_	h	Average time spent on the vibration process. It also includes the average time spent on transporting the GAR to the short curation area
*T*_*SC*_	h	Short curing time. It should be the minimum value that ensures that the demoulding process can be carried out with a low probability of breakage
*T*_*D*_	h	Average time spent on the demoulding process. It also includes the average time used for transporting each GAR unit to the long curation area
*T*_*LC*_	h	Long curing time. It should be the minimum value that ensures that the following processes can be carried out with a low probability of breakage
*T*_*LA*_	h	Average time spent on transporting each GAR unit to the loading area
*W*_*DM*_	days/week	Number of working days per week for the manufacturing process
*H*_*SM*_	–	Starting time of the working day for the manufacturing process
*H*_*EM*_	–	End time of the working day for the manufacturing process
*N*_*MGAR*_	GAR units	Minimum number of GAR units that should be available in the loading area so that the ground transport starts
*N*_*T*_	truck	Number of trucks that participate in the inland transport stage
*N*_*GART*_	GAR units	Maximum number of GAR units that can be loaded onto each truck
*C*_*T*_	€/day truck	Daily rent price of each truck including the driver
*T*_*CO2*_	kg of CO_2-eq._/km	Average CO_2-eq._ emission factor for each truck. Since it is an average parameter, the same value is used for the round journey
*D*_*LT*_	km	Average land transport distance
*S*_*T*_	km/h	Average land transport speed for the round journey
*T*_*L*_	h/GAR unit	Average time spent in loading one GAR unit onto the truck
*T*_*U*_	h/GAR unit	Average time taken to unload each GAR unit from the truck
*W*_*DLT*_	days/week	Number of working days per week for the ground transport process
*H*_*SLT*_	–	Starting time of the working day for the land transport process
*H*_*ELT*_	–	End time of the working day for the inland transport process
*Ns*	ship	Number of ships used for the sea transport
*N*_*GARS*_	GAR units	Maximum number of GAR units that can be loaded onto each ship
*C*_*S*_	€/day	Daily rent price of each ship
*S*_*CO2*_	kg of CO_2-eq._/Nm	Average CO_2-eq._ emission factor for each ship during transport when it is full of GAR units
*SB*_*CO2*_	kg of CO_2-eq._/Nm	Average CO_2-eq._ emission factor for each ship during ballast navigation
*SL*_*CO2*_	kg of CO_2-eq._/h	Average CO_2-eq._ emission factor for each ship during the loading process of GAR units
*SI*_*CO2*_	kg of CO_2-eq._/GAR unit	Average CO_2-eq._ emission factor for the installation of one GAR unit in its final location. It is an average value since there will be cases where the ship must reposition itself
*D*_*ST*_	Nm	Average sea transport distance
*S*_*T*_	kn	Average sea transport speed for the round journey
*TS*_*L*_	h/GAR unit	Average time spent in loading one GAR unit onto the ship
*TS*_*I*_	h/GAR unit	Average time taken to install each GAR unit in its final location. Again, there will be cases where ship must reposition itself
*W*_*DST*_	days/week	Number of working days per week for the sea transport process
*H*_*SST*_	–	Starting time of the working day for the sea transport process
*H*_*EST*_	–	End time of the working day for the sea transport process
*P*_*EP*_	€/GT	Port entry fee for each ship. It depends on its gross tonnage
*P*_*MP*_	€/m·day	Daily mooring cost for each ship. It depends on its length
*P*_*CP*_	€/t	Cost of loading GAR units at port

For the case study described in Section "Case study", the decision variables will be both the number of moulds (*N*_*M*_) and the number of trucks (*N*_*T*_). There will be other parameters that without being decision variables per se will vary depending on the option considered for the offshore stage (in particular, for LNG workboat, diesel barge, and electric specific design barge). Furthermore, intermediate calculations will be necessary for defining the value of some of the parameters included in Table 1. More information is provided in Section "Case study". With the exceptions previously mentioned, once both the parameters and the decision variables adopt specific values, AGARDO automatically provides the results in terms of total cost, time and equivalent CO_2_ emissions. It is important to note that the time spent on each activity is used, in certain cases, to estimate emissions and costs, as can be deducted from the units of measurement of some of the parameters included in Table 1. AGARDO also provides the number of round travels that both the trucks and the ship need for completing the whole process. The number of trips serves to estimate the total distance travelled by the vehicles and, consequently, the corresponding emissions of CO_2-eq._ (by using the emission factors included in Table 1). More information about emissions (sea transport stage) and costs is provided in Sections "Sea transport stage" and "Cost analysis" respectively.

## Case study

### Ares-Betanzos estuary

**Figure 3:**
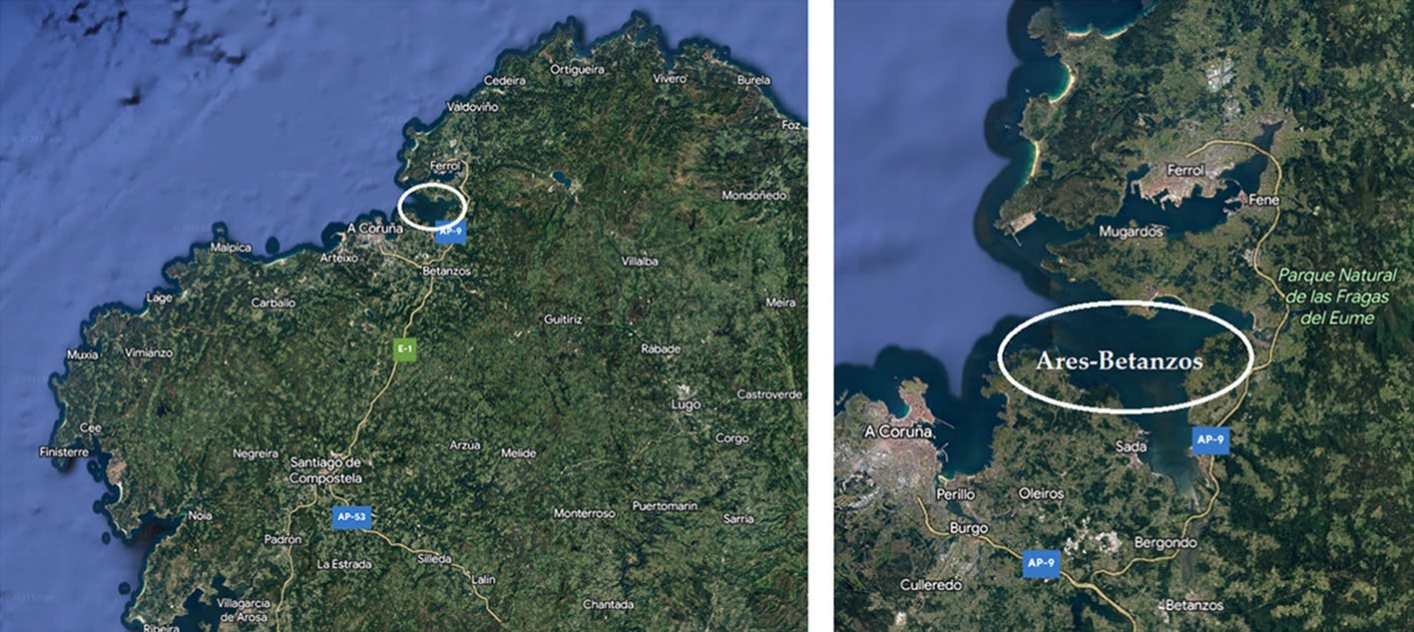
Location of the Ares-Betanzos estuary^47^. Source: own elaboration using Google Maps, 2021 version (https://www.google.es/maps).

**Figure 4:**
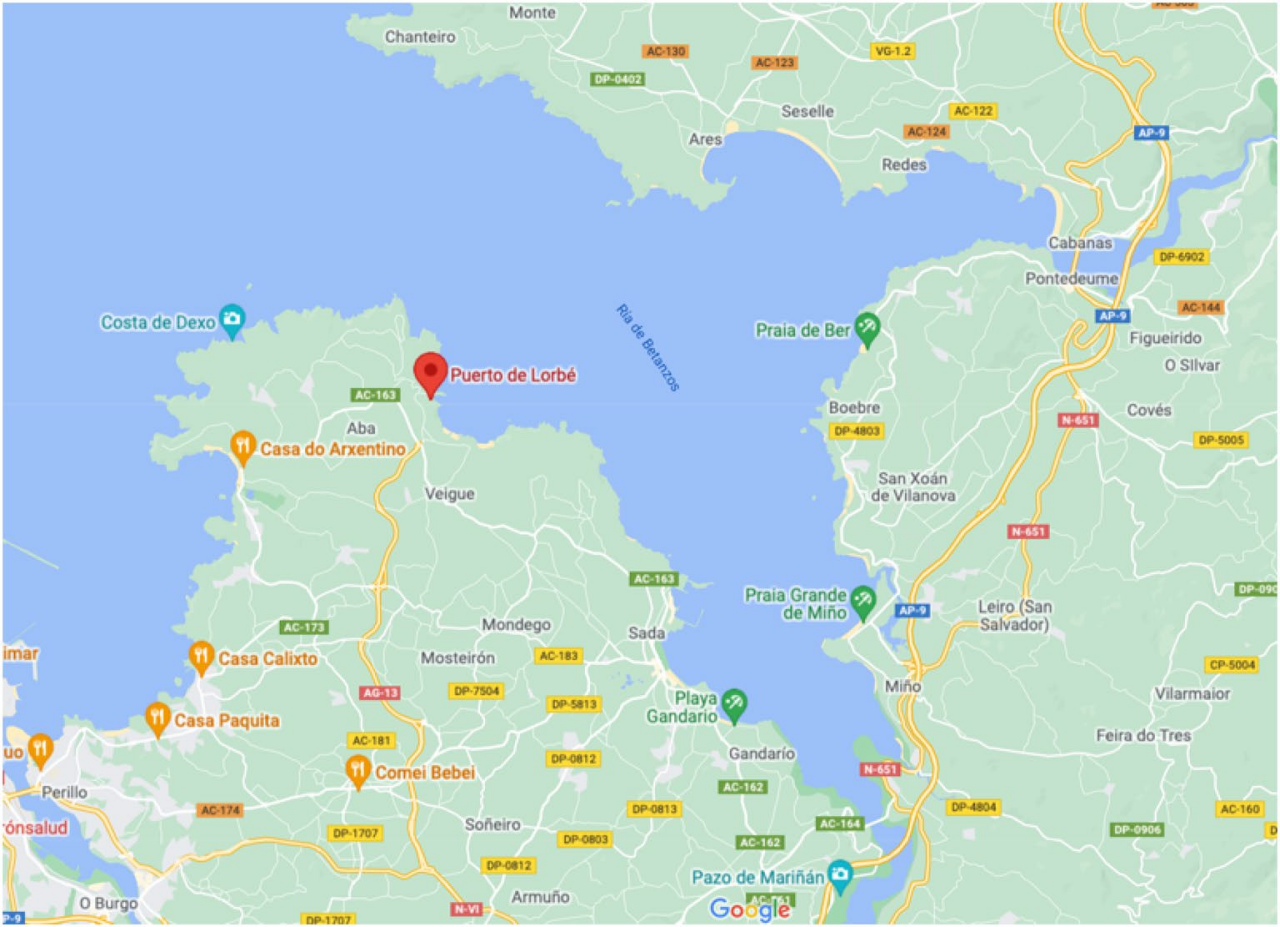
Port of Lorbé located in Oleiros (A Coruña)^47^. Source: own elaboration using Google Maps, 2021 version (https://www.google.es/maps).

The Ares-Betanzos estuary (Galicia, North-West of Spain, Fig. 3), also known as Ares-Betanzos ría, was selected as the case study to test AGARDO, being Lorbé the corresponding deployment port (Oleiros, A Coruña, Fig. 4). For the road transport stage, the first type of truck is a rigid one with three axles and a maximum permissible load capacity of 26 tonnes (t). The second one is an articulated truck with five axles and a maximum load capacity of 38 tonnes (t). The proposed values for some of the parameters associated with the manufacturing and inland transport stages will always be the same. Nevertheless, some of the parameters linked to the transport processes will vary depending on the type of truck or ship employed (Section "Sea transport stage"). The values adopted by the common parameters can be found in Table 2. The values adopted by the parameters affected by the type of truck used in the road transport are included in Table 3. It is important to note that average values were considered for the CO_2-eq._ emissions. Nevertheless, this is a simplified version of the reality, since emissions vary with the engine regime. To consider how the engine operation speed affects the emissions is out of the scope of this paper, although it could be included in a future version of the AGARDO tool.

**Table 2:** Values defined for the common parameters.

Parameter	Value	Units
*N*_*GAR*_	180	GAR units
*N*_*B*_	18	GAR units
*P*_*NM*_	100	–
*C*_*M*_	8000	€/mould
*T*_*P*_	1	h
*T*_*V*_	1	h
*T*_*SC*_	72	h
*T*_*D*_	3	h
*T*_*LC*_	96	h
*T*_*LA*_	0.25	h
*W*_*DM*_	5	days/week
*H*_*SM*_	8	–
*H*_*EM*_	18	–
*N*_*MGAR*_^*1*^	117	GAR units
*D*_*LT*_	160	km
*S*_*T*_	80	km/h
*T*_*L*_	0.25	h/GAR unit
*T*_*U*_	0.25	h/GAR unit
*W*_*DLT*_	5	days/week
*H*_*SLT*_	8	–
*H*_*ELT*_	19	–
*Ns*	1	ship
*D*_*ST*_	3	Nm
*W*_*DST*_	5	days/week
*H*_*SST*_	8	–
*H*_*EST*_	22	–

^1^Estimated as 65% of *N*_*GAR*_*.*

**Table 3:** Values defined for the parameters affected by the type of truck.

Parameter	Rigid truck	Articulated truck	Units
*N*_*GART*_	3	4	GAR units
*C*_*T*_	250	400	€/day truck
*T*_*CO2*_^1^	1.37	1.43	kg of CO_2-eq._/km

^1^Values taken from Carral et al.^26^.

The values that some of the parameters for the manufacturing process (*N*_*B*_, *T*_*P*_, *T*_*V*_, *C*_*M*_, *T*_*SC*_, *T*_*D*_, *T*_*LC*_ and *T*_*LA*_) adopt are based on the information provided by local concrete manufacturers. They must be understood as approximate values, being the order of magnitude more relevant than the exact figures included in Table 2. Furthermore, these values could vary depending on aspects such as the country where the GAR units are produced, the design of each GAR unit, the technologies and equipment used, the exact layout of the equipment in the manufacturing plant, among many others. The values for some of the parameters in Table 2 are specific of the case study under consideration. A case in point is the number of GARs to be installed (*N*_*GAR*_) or the transport distances (*D*_*LT*_ and *D*_*ST*_). Reasonable values were proposed for other parameters, such as the number of working days per week (*W*_*DM*_, *W*_*DLT*_, *W*_*DST*_) and the starting and end times for the working days (*H*_*SM*,_ *H*_*EM*_, *H*_*SLT*_, *H*_*ELT*_, *H*_*SST*_, *H*_*EST*_). Nevertheless, they could be modified to simulate alternative scenarios.

### Sea transport stage

Three different ships were considered for the offshore phase. The first one is a conventional barge using diesel as fuel. Barges are a kind of floating platforms with a steel structure. They are characterised by: a considerable load capacity (diaphanous deck for cargo), low cost of production, speed in its construction and start-up, low maintenance cost and, due to their geometry, they have considerable manoeuvrability, although it is reduced in comparison with other smaller ships. The one considered in this study is equipped with one crane for loading and unloading processes.

The second type of vessel is a LNG-fuelled workboat. There is a wide range of workboats in the market, with different load capacities, auxiliary systems (such as cranes or winches), powers and speeds. In general, they stand out for their great positioning flexibility compared to other conventional alternatives, typically counting on azimuth thrusters and with the possibility of making temporary adaptations on deck. Furthermore, the one considered here is also equipped with two cranes for loading and unloading the GAR units.

In terms of energy efficiency and with the objective of reducing the potential environmental impacts, the third alternative is an electric barge specifically designed for Galician estuaries. Consequently, it presents several advantages such as high manoeuvrability, and the possibility of operating in areas of very low draft. The detailed design of this option is out of the scope of this paper. Nevertheless, this barge must be designed in such a way it has enough space for the electric motor, the battery (or batteries), the cabin, and the auxiliary transport elements, among others. It will be equipped with a gantry crane. It will only be used for the artificial reef installation process. For the port loading activity, a crawler crane will be employed, in particular a LR 1110 Liebherr model^48^. The reader can find in Table 4 the most relevant characteristics of these three alternatives as well as the values adopted by the derived parameters. Appendix A includes basic schemes for the three ships proposed in this paper.

**Table 4:** Main characteristics of the three ships and values adopted by the corresponding AGARDO parameters.

Parameter (units)	Diesel-fuelled barge	LNG-fuelled workboat	Electric barge
*Ns* (ship)	1	1	1
Length (m)	34	26	25
Breadth (m)	27	11.5	13
Depth (m)	2.5	4	3
Draft (m)	1.8	2.7	1
Deck area (m^2^)	700	180	180
Deadweight tonnage (t)	1040	286	156
Gross tonnage (GT)	648	159	156
Main engine power, *P*_*ME*_ (kW)^1^	300	1300	200
Auxiliary engine power, *P*_*AE*_ (kW)	350	400	–
*S*_*T*_ (kn)	6	10	10
Number of cranes (crane)	1	2	1
Crane capacity (t)	9	9	9
Crane range (m)	35	10	–
Main engine load factor during navigation, *LF*_*ME*_	0.9	0.9	0.8
Main engine load factor during ballast navigation, *LF*_*ME*_	0.8	0.8	0.8
Main engine load factor during installation, *LF*_*ME*_	0	0	0.7
Auxiliary engine load factor during sea transport, *LF*_*AE*_	0	0.5	–
Auxiliary engine load factor during the loading process, *LF*_*AE*_	0.9	0.9	–
Auxiliary engine load factor during installation, *LF*_*AE*_	0.9	0.9	–
*N*_*GARS*_ (GAR units)	200	55	15
*C*_*S*_ (€/day)^2^	3800	5000	2140
*S*_*CO2*_ (kg of CO_2-eq._/Nm)	30.61	54.02	6.97
*SB*_*CO2*_ (kg of CO_2-eq._/Nm)	27.24	48.89	6.97
*SL*_*CO2*_ (kg of CO_2-eq._/h)	239.15	141.94	32.69
*SI*_*CO2*_ (kg of CO_2-eq._/GAR)	59.79	23.66	10.16
*TS*_*L*_ (h/GAR unit)	1/15	1/30	1/20
*TS*_*I*_ (h/GAR unit)	1/4	1/6	1/6
*P*_*EP*_ (€/GT)	3.75	3.75	3.75
*P*_*MP*_ (€/m day)^3^	1.02	1.02	1.02
*P*_*CP*_ (€/t)	1.24	1.24	1.24

^1^There is a considerable difference in the main engine power of each ship. This is due to their different sizes, designs and gross tonnage. On the other hand, it is important to note that the data for the diesel-fuelled barge and for the LNG-fuelled workboat are based on real, existing ships, while the data for electric barge were specifically conceived for this case study.

^2^Based on^49^. In the particular case of the electric barge, more information is provided in section "Cost analysis".

^3^Based on^50^.

In the existing literature, there are different ways of estimating the equivalent CO_2_ emissions (*S*_*CO2*_, *SB*_*CO2*_, *SL*_*CO2*_ and *SI*_*CO2*_) during the different stages of the offshore phase. In this line, it is important to distinguish between the two alternatives using fossil fuels (barge and workboat) and the electric vessel.

For the diesel and LNG fuelled alternatives, the emission of the following pollutants was considered: CO_2_, N_2_O, CH_4_ and BC (black carbon), as they are some of the most relevant ones in terms of global warming potential (GWP) in engines^51^. Equation (1) was used for estimating the amount in kg (*E*_*i,j*_) for CO_2_, N_2_O, CH_4_ and BC, with the exception of the latter for the diesel barge. It is a proposal based on Olmer et al.^51^.

1 $${E}_{i,j}=\frac{1}{1000}\cdot \left({P}_{ME}\cdot {LF}_{{ME}_{i}}\cdot {EF}_{{ME}_{j}}+{P}_{AE}\cdot {LF}_{{AE}_{i}}\cdot {EF}_{{AE}_{j}}\right)\cdot {T}_{i}$$

where *i* denotes the stage (navigation, ballast navigation, loading process or installation) and *j* the pollutant. *EF*_*Mej*_ is the main engine emission factor for pollutant *j* and its units of measurement are g/kWh. *EF*_*AEj*_ is a similar parameter but, in this case, for the auxiliary engine. Finally, *T*_*i*_ is the time in hours associated with the same operation conditions. It has been assumed that both the main engine and the auxiliary engine present a constant operating condition during each stage, as can be deducted from Table 4. Once again, this is a simplification of the reality, since the operating conditions of an engine can vary instantaneously. Therefore, for each stage, the load factors should be understood as average values. Equation (1) can be easily modified for changing operating conditions, by including a summation that accounts for each hour under the same conditions. The reader can find the values for *EF*_*MEj*_ and *EF*_*AEj*_ in Table 5^51^.

**Table 5:** *EF*_*MEj*_ and *EF*_*AEj*_ values for the different pollutants and ships^51^.

Emission factor	Diesel-fuelled barge			LNG-fuelled workboat			
	CO_2_	N_2_O	CH_4_	CO_2_	N_2_O	CH_4_	BC
*EF*_*MEj*_ (g/kWh)	658	0.03	0.01	366	0.01	0.94	2E−3
*EF*_*AEj*_ (g/kWh)	696	0.03	0.01	366	0.01	0.94	2E−3

The amount of BC in kg emitted by the diesel-fuelled barge is estimated through Eq. (2)^51^.

2 $${E}_{i,BC}=\frac{1}{1000}\cdot \left({FC}_{{ME}_{i}}\cdot {EF}_{{ME}_{BC}}+{P}_{AE}\cdot {LF}_{{AE}_{i}}\cdot {EF}_{{AE}_{BC}}\right)\cdot {T}_{i}$$

where *FC*_*MEi*_ is an approximation of the fuel consumption for the main engine, measured in kg. It is estimated throughout Eq. (3)^51^. *EF*_*MEBC*_ is the emission factor associated with the main engine for BC, measured in g/kg of fuel and it depends on the load factor (Table 6). Similarly, *EF*_*AEBC*_ is the emission factor for the auxiliary engine measured in g/kWh and it adopts a value of 0.06 for the particular case here analysed^51^.

**Table 6:** BC emission factor for the main engine^51^.

Emission factor	Navigation (*LF*_*ME*_ = 0.9)	Ballast navigation (*LF*_*ME*_ = 0.8)
*EF*_*MEBC*_ (g of BC/kg fuel)	0.14	0.15

3 $${FC}_{{ME}_{i}}=\frac{{E}_{i,{CO}_{2}}}{{CI}_{f}}$$

In Eq. (3) *E*_*i,CO2*_ is the amount of CO_2_ emitted during stage *i* and it is calculated by employing Eq. (1). *CI*_*f*_ is the CO_2_ intensity of fuel *f* and it adopts a value of 3.206 (kg CO_2_/kg of fuel) for this study^51^. With the amount emitted of each one of the 4 pollutants and their corresponding characterisation factors (*CF*_*j*_, measured in kg of CO_2-eq._/kg of pollutant *j*), it is possible to estimate the corresponding equivalent CO_2_ emissions for each one of the stages *i* (*S*_*i*_ measured in kg of CO_2-eq._), Eq. (4). The reader can find in Table 7 the corresponding characterisation factors.

**Table 7:** Characterisation factors for 100-year GWP^51^.

Characterisation factor	Pollutant *j*			
	CO_2_	CH_4_	N_2_O	BC
*CF*_*j*_ (kg CO_2-eq._/kg of j)	1	25	298	900

4 $${S}_{i}=\sum_{j}{CF}_{j}\cdot {E}_{i,j}$$

Finally, *S*_*CO2*_, *SB*_*CO2*_, *SL*_*CO2*_ and *SI*_*CO2*_ are calculated from the different *S*_*i*_ values. In order to do so, it is necessary to take into account parameters such as *TS*_*L*_, *TS*_*I*_, *D*_*ST*_ or *S*_*T*_ among others, to achieve the units of measurement indicated in Table 1. In this line, the reader should bear in mind that *S*_*CO2*_, *SB*_*CO2*_, *SL*_*CO2*_ and *SI*_*CO2*_ could have been expressed in other units. By way of example, *S*_*CO2*_ and *SB*_*CO2*_ could be calculated for each journey instead of being expressed per nautical mile. Similarly, *SL*_*CO2*_ could have been measured in kg of CO_2-eq._ per GAR unit. In fact, the units of measurement depend on how AGARDO is programmed and the information it saves during the simulation. Nevertheless, the reader can find in this paper all the necessary information to modify the units of measurement of these parameters.

The estimation of the emission factor for the electric barge is not done through Eq. (1–4). For the loading process, *SL*_*CO2*_ is calculated by using Eq. (5), resulting in a value of 32.69 kg of CO_2-eq_/h.

5 $${SL}_{CO2}={F}_{C}\cdot EF$$

In Eq. (5), *F*_*C*_ is the average consumption of the crawler crane measured in l/h when it is in operation. It adopts a value of 8.47 based on the information provided by Liebherr Group^52^. *EF* is the average emission factor for diesel vehicles measured in kg of CO_2-eq._ per litre. Its value is 3.86 and it is based on the information provided by Bicer and Dincer^53^.

For the calculation of *S*_*CO2*_, *SB*_*CO2*_ and *SI*_*CO2*_, it is first necessary to make an approximate estimation of battery type and size. In this line, a Lithium-ion battery has been chosen. Its sizing has been carried out taking as reference the study performed by Roland et al.^54^. The following considerations have been made: (i) 15% of the energy is lost through the drive chain, (ii) a safety margin of 20% was established, (iii) only 60% of the installed capacity can be used, and (iv) the efficiency of the chargers is about 85%^54^. The main engine power of the electric barge is 200 kW (Table 4). Taking into account its speed and the distance (Table 4), the electric barge needs 0.6 h to complete the transportation stage. With a load factor of 0.8, 96 kWh will be consumed. Regarding the installation stage, the electric barge needs 2.5 h to position the maximum number of GARs it can carry. During this process, the load factor is 0.7 (Table 4), resulting in 350 kWh being consumed. With these two values and taking into account the previous considerations, it would be necessary to install a 1026 kWh battery pack.

It is also necessary to estimate the consumption in kWh for the different stages by multiplying *P*_*ME*_ by the time (h) consumed in each one of them. To this must be added the corresponding losses as well as the chargers’ efficiency. The resulting values in kWh must then be multiplied by the average CO_2-eq._ emissions from the production of 1 kWh. A value of 0.3218 kg CO_2-eq._/kWh was adopted^26,55^. It can be considered as a realistic and probable value for the Spanish electricity grid mix. On the other hand, the comment on the units of measurement made for the other two ships is also valid for this case.

In the same way that this section includes detailed information on emissions, Section "Cost analysis" provides additional data on the costs.

### Cost analysis

AGARDO estimates the total cost of the process (*TC*, measured in €) through Eq. (6). As previously explained the costs associated with both raw material procurement and manufacturing processes are not included in this study.

6 $$TC={C}_{TM}+{C}_{LT}+{C}_{ST}$$

where *C*_*TM*_, *C*_*LT*_ and *C*_*ST*_ are the total costs associated with the moulds, the land transport stage and the sea transport phase, respectively, all of them measured in €. *C*_*TM*_ is obtained by using Eq. (7):

7 $${C}_{TM}={N}_{M}\cdot {C}_{M}$$

The cost of the road transport (*C*_*LT*_) is determined by Eq. (8).

8 $${C}_{LT}={N}_{T}\cdot {C}_{T}\cdot {D}_{RT}$$

where *D*_*RT*_ is the number of days that each truck is rented and it is one of the outputs provided by AGARDO. *C*_*T*_ (Table 3) includes driver, fuel and financial costs, as well as the amortisation, the truck maintenance cost and the corresponding insurance.

On the other hand, the cost of the sea transport stage (*C*_*ST*_) is made up of two distinct costs (Eq. (9)): (i) the cost of renting the ships (*C*_*RS*_), and (ii) the cost associated with port activities (*C*_*PA*_).

9 $${C}_{ST}={C}_{RS}+{C}_{PA}$$

*C*_*RS*_ is obtained from Eq. (10).

10 $${C}_{RS}={N}_{s}\cdot {C}_{s}\cdot {D}_{RS}$$

*D*_*RS*_ is an analogous parameter to *D*_*RT*_ but, this time, for ships. It is also a result returned by AGARDO. In the particular case of the electric barge, as it is specifically designed for the case study analysed in this paper, its value for *C*_*S*_ was estimated as the minimum value (in €/day) that makes the net present value equal to zero. For such a purpose, an initial investment for the electric barge of 566,101.87 € was considered. This initial investment is distributed in two years, the time spent in the construction. A residual value of 5% was also assumed. Furthermore, the electric barge has been considered to operate for 100 days per year with a service life of 20 years. A value of 7% was established for the capital cost. To this must be added the costs associated with the crew (4 workers with an average salary of 28,000 €/year) as well as the costs derived from the electricity consumed in charging the battery. In this regard, a price of 0.18 €/kWh has been used. All of the above results in a *C*_*S*_ value of 2140 €/day (Table 4). Furthermore, the propulsion cost was calculated taking into account the cost of the electric engine, the battery pack and the ancillary systems. This cost is more than 300 thousand euros; the battery pack is the greatest expense because it represents around 200,000 euros.

On the other hand, *C*_*PA*_ depends on three main factors: (i) port entry, (ii) mooring, and (iii) cargo. The cost of port entry *C*_*PE*_ is estimated by using Eq. (11). It is based on the Galician port law^50^.

11 $${C}_{PE}={N}_{s}\cdot \left[\left({N}_{ST}+1\right){\cdot P}_{EP}\cdot \frac{Gross tonnage}{100}\right]$$

In Eq. (11), *N*_*ST*_ is the number of round travels for the ship and it is provided by AGARDO. In this case study, there is only one value for *N*_*ST*_, since only one ship is used in each scenario (*N*_*s*_ = 1). However, Eq. (11) can be easily modified for those cases in which more ships are employed.

The mooring cost (*C*_*MOOR*_) was also estimated according to the Galician port law^50^. In Eq. (12), *T*_*MOOR*_ is the time in days that each ship is docked in port and it is also returned by AGARDO.

12 $${C}_{MOOR}={N}_{s}\cdot {T}_{MOOR}{\cdot P}_{MP}\cdot Length$$

The cargo cost (*C*_*CARGO*_) was calculated by applying Eq. (13)^50^, where 5 is the number of tons of each GAR unit.

13 $${C}_{CARGO}=5\cdot {N}_{GAR}\cdot {P}_{CP}$$

*C*_*PA*_ is calculated through Eq. (14).

14 $${C}_{PA}={C}_{PE}+{C}_{MOOR}+{C}_{CARGO}$$

Finally, it is important to remark that the electric barge presents an additional cost derived from the use of the crawler crane indicated in Section "Sea transport stage", for port activities.

The decision variables considered in this case study are *N*_*M*_ (number of moulds) and *N*_*T*_ (number of trucks). Both are integer numbers. *N*_*M*_ adopts odd values between 5 and 15, while *N*_*T*_ varies from 1 to 4. The scenarios resulting from all the possible combinations have been simulated resulting in a total number of 144 simulations (two different types of trucks as well as three different ships).

## Results and discussions

Due to length constraints, it is not possible to present and discuss in depth all the results. Consequently, only the most relevant ones according to the main objectives of the paper are included in this section. The reader can find in Appendix A more information. In this sense, it is important to note that, with the AGARDO tool, different scenarios were generated. In particular, they were obtained from the variation in the number of trucks, type of truck, type of ship and number of moulds. Therefore, for every type of truck and ship, each scenario analyses the results for a varying number of moulds (5–15).

Regarding the total time (in days) employed for the manufacture, transport and installation processes, significant differences were not found among the three types of vessels considered in this study, Table 8.

**Table 8:** Minimum and maximum time spent in the whole process.

Type of vessel	Minimum time (days)	Maximum time (days)
Diesel barge	58	142
LNG workboat	51	135
Electric barge	50	134

The type of truck used for the ground transport has little effect on the total time. In other words, once the vessel type and the number of moulds have been established, no major differences have been found between using the rigid and the articulated truck. In fact, the biggest difference is about two days. On the other hand, the same trend is always observed for each type of vessel: increasing the number of trucks while keeping the number of moulds constant hardly reduces the total time. That is, for the same number of moulds, the difference between using one or four trucks reaches a maximum value close to one day. Therefore, the results suggest that both the type and the number of trucks are not relevant in terms of timescales. Consequently, ignoring the impacts that the trucks cause on the total time, it is possible to say that the largest difference among the three types of vessel is ten days (keeping the number of moulds constant). The impact of vessel type on time is greater than that generated by trucks. Despite this, it is still not relevant compared to the differences generated by the number of moulds. This confirms that the number of moulds is the real bottleneck of the process and, therefore, the most relevant parameter for timescales. The selection of the type of vessel and truck (as well as the number of the latter) should be derived from economic or environmental (equivalent CO_2_ emissions) results.

As for the number of moulds, similar behaviour has been observed in the impact it has on time. That is, increasing the number of moulds by two units leads to almost the same reduction in time, regardless of the type of vessel used, Table 9.

**Table 9:** Reduction in the number of days caused by the increase in the number of moulds.

Initial number of moulds	Final number of moulds	Time reduction (days)
5	7	36–38
7	9	18–20
9	11	13–14
11	13	7–8
13	15	6–7

From Table 9, it is clear that as the number of moulds increases, the reduction in time this produces becomes smaller. There will be a value beyond which it is no longer worthwhile to increase the number of moulds, since the reduction in the number of days will be negligible. The reader should note that the same trend is expected if the number of moulds is increased by one, rather than by two.

It is important to note that weather conditions can be really adverse on the Galician coast. The best months of the year for offshore operations are July and August. In this regard, it would be desirable to complete the entire process within a period of around 60 days. This implies that, with the options analysed in this study, the number of moulds should vary between 13 and 15 moulds (even 11 for the workboat and the electric barge).

**Figure 5:**
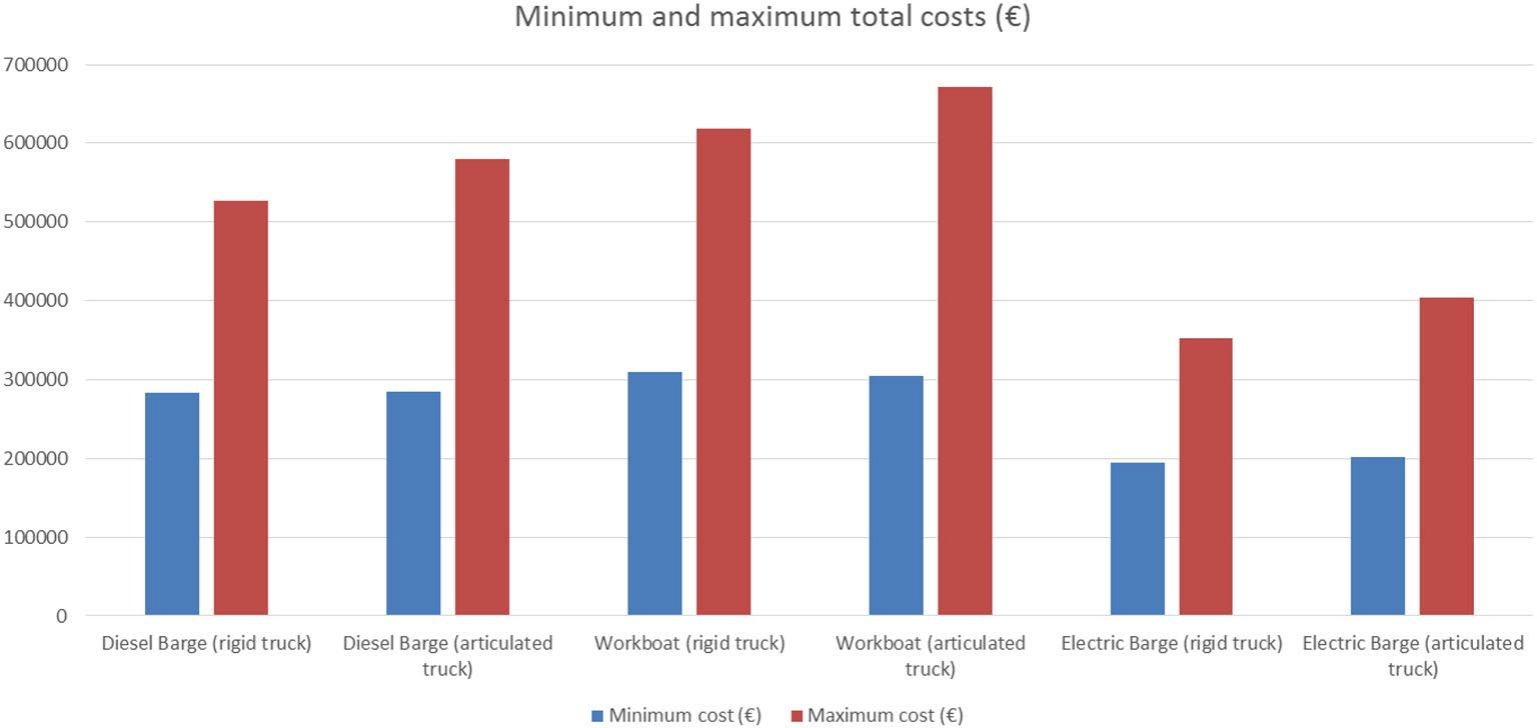
Minimum and maximum costs for each one of the vessels. Source: own elaboration.

Looking at the costs, relevant differences have been obtained among the simulated alternatives (Fig. 5). In fact, the worst option (workboat and articulated truck) implies a cost overrun of more than 475,000 € compared to the best one (electric barge and rigid truck).

Figure 5 shows that the electric barge is the most economical option, followed by the diesel-fuelled barge and by the LNG-fuelled workboat, respectively. A more detailed analysis of the 144 simulated scenarios (Appendix A) always leads to the same conclusion. In particular, if costs are analysed when the number of moulds varies between 11 and 15 (and once both the type and number of trucks are established), the electric barge is always the best option followed by the diesel barge and the workboat. However, the reader should bear in mind that the results for the electric barge were obtained by assuming a price of 0.18 €/kWh. Therefore, once the best option has been determined from an economic point of view, it is necessary to analyse how the type of truck as well as the number of trucks and moulds affect the costs.

For a given type of ship, once the number of moulds adopts a specific value, it is observed that as the number of trucks increases (regardless of whether they are rigid or articulated), the total cost also increases. This logical result coupled with the fact that the number of trucks hardly affects the total time, suggest that the best option is to hire a single truck. As for the type of truck, once a specific ship has been selected and the number of moulds has been set, the rigid truck resulted to be more economical than the articulated one in 143 out of 144 scenarios. The use of articulated trucks can lead to an extra cost over 50,000 €, with the smallest difference reaching a value close to 2,220 €. In the only case in which the articulated option presented a better performance, the difference between the two types of trucks adopted a value of about 4000 €. However, it is important to remark that this is a direct consequence of the figures established for *C*_*T*_ in Table 3. These values are appropriate for the particular case of Galicia, where the use of rigid trucks still competes with the articulated alternative. In other words, the daily cost of both options could be similar in other regions or even in other countries, so that the articulated option should outperform the rigid one.

**Figure 6:**
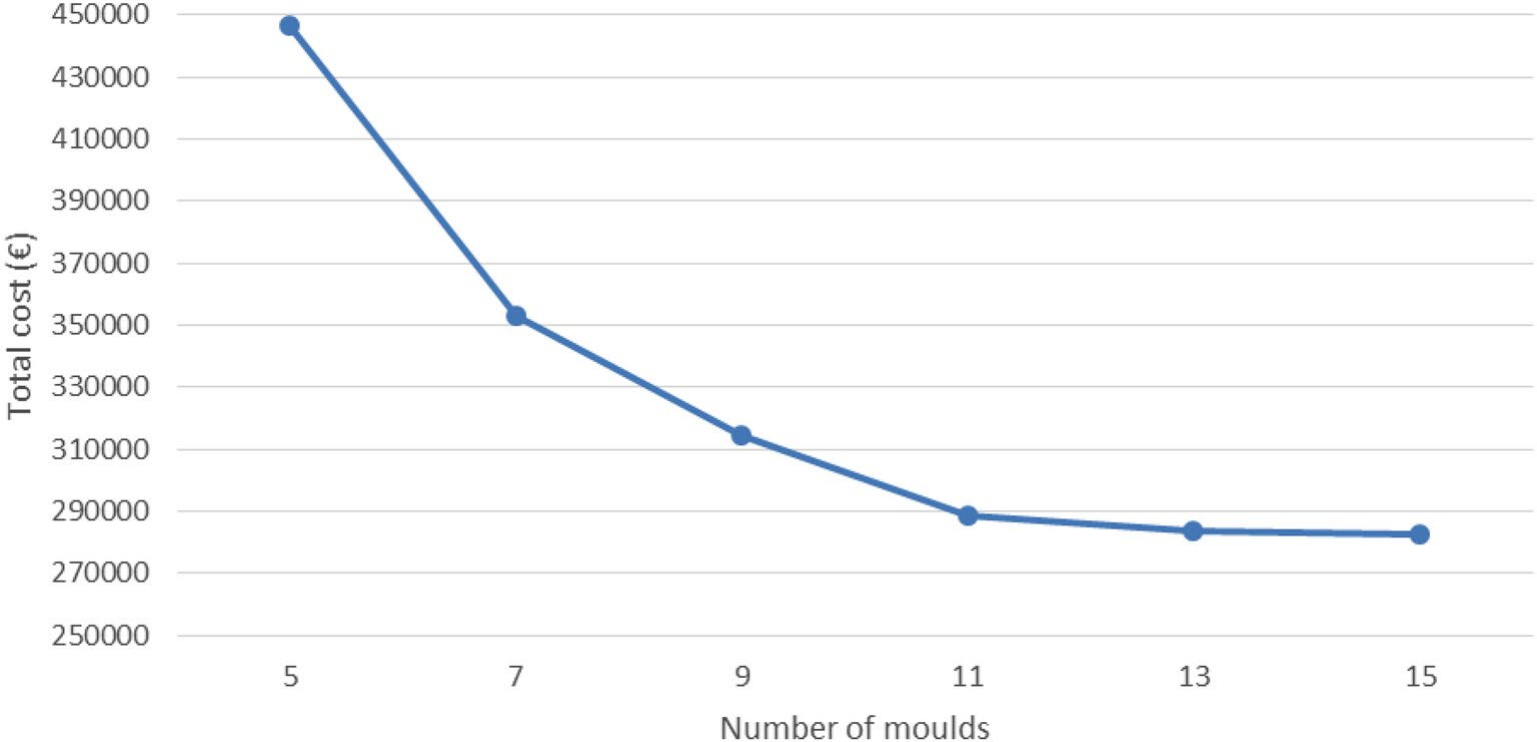
Evolution of costs versus the number of moulds for the diesel barge with only one rigid truck. Source: own elaboration.

Regarding the number of moulds, it has been observed that for a given type of vessel and truck and also for a given number of trucks, an increase in the number of moulds usually generates a decrease in the total cost (only 10 exceptions out of 144 scenarios, where the total cost hardly changes). This is a consistent result derived from what has already been discussed in terms of time. A higher number of moulds implies a reduction in the number of days required for completing the entire process. This, in turn, means that both the ship and the trucks will be hired for fewer days, which also reduces costs. Similar to what has been explained for timescales, as the number of moulds increases, the reduction in costs (in monetary values) usually decreases. By way of example, Fig. 6 shows the evolution of costs versus the number of moulds for the particular case of diesel barge with only one rigid truck. Similar graphs for other scenarios can be constructed from the data included in Appendix A. On the other hand, it should be emphasised that increasing the number of moulds serves both to achieve better results in terms of timescales and from an economic point of view. At this point, AGARDO supports the use of an electric barge with only one rigid truck and 11 moulds or more for the case study here addressed. Nonetheless, before taking a final decision, it is necessary to analyse the environmental results (CO_2-eq._ emissions).

**Figure 7:**
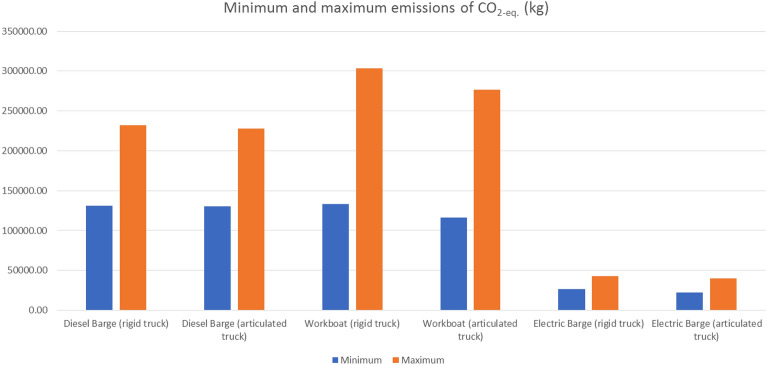
Minimum and maximum total emissions of CO_2-eq._ for each one of the vessels. Source: own elaboration.

Analogous to what happened for costs, relevant differences have been found for the total emissions, Fig. 7. Once again, the electric barge resulted to be the best option, with a considerable difference compared to the other two alternatives. In fact, if all scenarios are taken into account (Appendix A), for the same type of truck, number of trucks and number of moulds, the electric barge always outperforms the other two ships. As expected, in almost all scenarios, the emissions generated during the sea transport stage are higher than that for the inland transport one (there is only one exception for the electric barge with rigid trucks). Despite this, there is a noticeable difference among scenarios. By way of example, for the diesel barge with articulated trucks, emissions from sea transport are between 7 and 25 times higher than the ones generated during the land transport stage. This range of variation is smaller for the electric barge.

In terms of how the type of truck affects the emissions, no clear pattern of behaviour has been detected. It is important to remark that the two trucks adopt different values for the parameters *N*_*GART*_ and *T*_*CO2*_ (Table 3). Therefore, scenarios have been identified in which the use of the articulated truck is associated with lower total emissions and also with lower emissions during the land transport stage. However, in other scenarios, the articulated truck produced fewer emissions during the transport phase, while the total amount of equivalent carbon dioxide emissions resulted to be higher than in the equivalent scenario with rigid trucks. Despite this, there are more scenarios in which the articulated option performs better than the rigid one from an environmental point of view. This is in conflict with the results obtained at the economic level for the particular case of Galicia. The reader should note that economic and environmental aspects are often competing objectives, so that an improvement in one usually comes at the cost of getting worse in the other and vice versa. Consequently, a compromise solution needs to be found.

It is now time to analyse how the number of trucks impacts on emissions (once the type of truck, type of ship and number of moulds are established). More trucks should not necessarily mean more emissions, since as the number of trucks increases, the number of trips made by each truck should suffer the opposite effect. In fact, in 136 of the simulated scenarios, an increase in the number of trucks caused land transport emissions to decrease or to remain constant. However, what is really relevant is to analyse the effect on the total emissions. In this line, in 120 scenarios, a higher number of trucks has been associated with either a decrease in total emissions or no change in the corresponding values. Therefore, the opposite effect was observed in only 24 cases. It is possible to state that, in most cases, an increase in the number of trucks means an improvement in the environmental performance. On the other hand, this trend could be reversed if the number of trucks takes a value greater than four. In other words, a large number of trucks operating at the same time can cause a negative impact on traffic intensity, leading to traffic jams and more variable operating conditions for the engines. This could lead to greater emissions. However, this is not the case with up to four trucks.

Regarding the number of moulds, no absolutely predominant tendency has been detected on its impact on the total emissions. In 65 occasions, a higher number of moulds served to increase the total emissions. Nevertheless, in the remaining ones, the opposite effect has occurred. In other words, increasing the number of moulds often has positive impacts in terms of costs and timescales, but can generate both positive and negative impacts on total emissions. By way of summary, the reader can find in Table 10 the best solution for each one of the three objectives (time, cost and emissions). Table 10 shows that the minimum number of days (50) is carried out considering 15 moulds and an articulated truck. Thus, the minimum cost was achieved considering rigid truck and 15 moulds and the minimum emissions were obtained for 11 moulds and 64 days.

**Table 10:** Best solution for each one of the three objectives (time, cost and emissions).

Objective	Type of ship	Type of truck	Number of moulds	Number of trucks	Total time (days)	Total cost (€)	Total emissions (kg CO_2-eq._)
Time	Electric	Articulated	15	3–4	50	240,480–257,680	39,598–36,900
Cost	Electric	Rigid	15	1	52	195,298	37,905
Emissions	Electric	Articulated	11	3–4	64	246,804–267,204	22,398

From the results obtained, it can be concluded that there is no single best solution in terms of time, costs and emissions. As a result, the decision-maker must select the strategy according to the objective to be prioritised. On the other hand, as already anticipated, another option is to define a compromise solution with a remarkable performance on all three objectives. Given the relevance of weather windows in Galicia, this solution should be sought among those scenarios that can be completed in 70 days or less. Ten combinations have been found with: (i) a total cost below 230,000 €, and (ii) total emissions of less than 35,000 kg CO_2-eq._, (Table 11). It would also be possible to look for compromise solutions between the other two types of ships, in case an electric barge is not available.

**Table 11:** Potential compromise solutions.

Type of ship	Type of truck	Number of moulds	Number of trucks	Total time (days)	Total cost (€)	Total emissions (kg CO_2-eq._)
Electric	Rigid	11	2	64	207,132	34,889.72
Electric	Rigid	11	3	64	222,804	29,215.73
Electric	Rigid	13	2	57	208,759	34,947.42
Electric	Rigid	15	3	51	223,796	33,683.32
Electric	Articulated	11	1	64	201,432	31,398.59
Electric	Articulated	11	2	64	225,204	24,775.17
Electric	Articulated	13	1	57	205,925	25,883.28
Electric	Articulated	13	2	57	223,759	28,911.87
Electric	Articulated	15	1	51	210,811	25,885.99
Electric	Articulated	15	2	50	225,446	24,616.48

Finally, a sensitivity analysis was done; it was based on the fluctuation in fuel prices. In this case study, the fluctuating price of fuel will affect the affreightment contract established for the vessel, which is defined inside AGARDO. Therefore, the sensitivity analysis has been carried out based on the variation in the price of this contract depending on the price of fuel. Observing how the prices of LNG, marine diesel and electricity have varied in the last three years, the most significant difference in the contract cost range occurs in the case of the LNG workboat. With this form of energy, the range goes up to 50% between low and high charter prices, while diesel and electricity do not reach 30%. In the worst scenario of high freight prices, the LNG workboat is the most expensive option, and the cheapest option is clearly the electric barge. However, in a low freight scenario, the difference in cost between the three options is not so significant. Therefore, in this case, the choice of the electric barge over the rest of the options will be based more on the criterion of minimising the carbon footprint than on the total operating costs. The reader can make a more detailed analysis of the results obtained from Appendix A, including the sensitivity analysis.

## Conclusions and future developments

This paper presents a new simulation tool called AGARDO (Automatic Green Artificial Reef Deploy Optimisation), for the production, transport and installation of artificial reefs in shallow waters. It was developed by using Python language as well as the SimPy library. AGARDO includes both onshore (manufacturing, road transport and unload at port) and offshore (load at port, sea transport, positioning, and deployment tasks) stages. It allows the user to simulate different scenarios, providing the following results: (i) total costs, (ii) number of days required to complete the whole process, and (iii) total emissions of equivalent carbon dioxide. AGARDO is a tool in which the user can easily introduce modifications. By way of example, it makes it possible to model different options for both land and sea transport stages. It also allows the user to modify the value that each parameter adopts.

This new methodology was applied to an estuary located in Galicia (North-West of Spain), where 180 artificial reef units must be installed. To solve this case study, three different options were analysed for the sea transport stage (diesel barge, LNG workboat, and an electric specific design barge), as well as two alternatives for inland transport (rigid and articulated truck). A total number of 144 scenarios have been generated, varying the type of sea and land vehicle, the number of trucks and the number of moulds employed during the manufacturing stage (artificial reef production).

AGARDO proved to be a valuable tool given that significant differences were found among the 144 scenarios, in terms of costs, timescales and emissions. From the results obtained, it is possible to say that no single best solution was found related to these three factors. In other words, the environmental objective is often in conflict with the economic one. Consequently, the decision-maker should select a compromise solution among the ones provided by AGARDO. For the case study analysed here, the compromise solution involves using an electric barge with between 11 and 15 moulds.

Going into greater detail, the number of moulds became the main bottleneck in the process. Furthermore, an increase in the number of moulds usually leads to a decrease in the total cost, although the monetary values are steadily decreasing. The same is not true at the environmental level, since an increase in the number of moulds can produce both positive and negative impacts on total emissions. When the different ships are compared, the electric barge turned out to be the most economical option, followed by the diesel-fuelled barge and LNG-fuelled workboat, respectively.

The electric barge also achieved the best environmental results, with a considerable difference in comparison with the other two alternatives. In the particular case of Galicia, where the rigid truck still competes with its articulated counterpart, the rigid option for inland transport proved to be more economical than the articulated one in 143 out of 144 scenarios. However, if daily costs from other regions were used, the opposite results would be obtained. Nevertheless, there are more scenarios in which the articulated truck obtained better environmental results than the rigid one. Moreover, in most cases, an increase in the number of trucks means improved environmental performance.

Regarding future developments, AGARDO can be applied to other estuaries. New options can be included in terms of both ground and sea transport. For instance, it would be possible to model in greater detail the varying conditions of the engines and their corresponding impacts on emissions. From the point of view of energy efficiency and reduced impact on the ecosystem, in the future the system’s motors may be totally electric, by adding a set or sets of interchangeable rechargeable batteries on land. This would mean that the vehicle could be operated without interruption. At a logistical level, the batteries do not have to pose any problem due to the fact that, in all cases, reef deployment is carried out within the vicinity of a reference port.

Although it is not relevant to this case study, it would be interesting to model how the number of trucks can affect traffic volume and thus emissions. Furthermore, it would be desirable to extend the sensitivity analysis, varying other parameters. Further improvement could also be made with a more detailed battery design and calculation. It would also be possible to extend the scope of the study by incorporating different options for the procurement of raw materials and also for the manufacturing stage. In fact, the model does not include the simulation of supply logistics. The manufacturing module starts assuming that there is unlimited availability of manufacturing materials, when, in reality, this is not always the case. Similarly, new environmental impact categories can be added such as acidification, eutrophication, and depletion of the ozone layer, among many others. Finally, from a methodological point of view, the most obvious improvement would involve the use of optimisation techniques, avoiding the simulation of hundreds or even thousands of scenarios.

### Supplementary Information

Supplementary Information.

## Data Availability

All data generated or analysed during this study are included in this published article (and its supplementary information files).
