# Supplementary material for: Land and sea transport options for the installation of green artificial reefs (GARs) in shallow waters: a Galician case study
Source: Sci Rep. 2024 Feb 1;14:2729. doi: 10.1038/s41598-024-53183-0 (PMC10834440; doi:10.1038/s41598-024-53183-0)
Supplement: Supplementary file 1 — Supplementary Information. [file 41598_2024_53183_MOESM1_ESM.docx]

**APPENDIX A:**

**Land and sea transport options for the installation of green artificial reefs (GARs) in shallow waters: A Galician case study**

Juan José Cartelle Barros ^a,*^, Alicia Munín-Doce ^b^, Laura Castro-Santos ^c^, Javier Lamas ^d^, Luis Carral ^e^

*^a^ Universidade da Coruña, Campus Industrial de Ferrol, CITENI, Departamento de Ciencias da Navegación e Enxeñaría Mariña, Escola Politécnica de Enxeñaría de Ferrol, Esteiro, 15471 A Coruña, Spain*

*^b^ Universidade da Coruña, Campus Industrial de Ferrol, CITENI, Departamento de Enxeñaría Naval e Industrial, Escola Politécnica de Enxeñaría de Ferrol, Esteiro, 15471 Ferrol, Spain*

*^c^ Universidade da Coruña, Campus Industrial de Ferrol, CITENI, Departamento de Enxeñaría Naval e Industrial, Escola Politécnica de Enxeñaría de Ferrol, Esteiro, 15471 Ferrol, Spain*

*^d^ Anta Norte, Lugar Avenida Mestre Manuel Gómez Lorenzo, 30, Vedra, 15885, A Coruña, Spain*

*^e^ Universidade da Coruña, Campus Industrial de Ferrol, CITENI, Departamento de Enxeñaría Naval e Industrial, Escola Politécnica de Enxeñaría de Ferrol, Esteiro, 15471 Ferrol, Spain*

^*^ Corresponding Author. Tel.: +34 650 93 90 53.

E-mail addresses: juan.cartelle1@udc.es (J.J. Cartelle Barros), a.munin@udc.es (A. Munín-Doce), laura.castro.santos@udc.es (L. Castro-Santos), javier.lamas@antanorte.com (J. Lamas), l.carral@udc.es (L. Carral).

ORCID: J.J. Cartelle Barros: 0000-0003-3139-5757. A. Munín-Doce: 0000-0002-0265-8805. L. Castro-Santos: 0000-0001-9284-1170. J. Lamas: 0000-0002-3497-9363. L. Carral: 0000-0003-1109-1131.

Appendix A provides additional information.

**A.1. MATERIALS AND METHODS: Additional information**


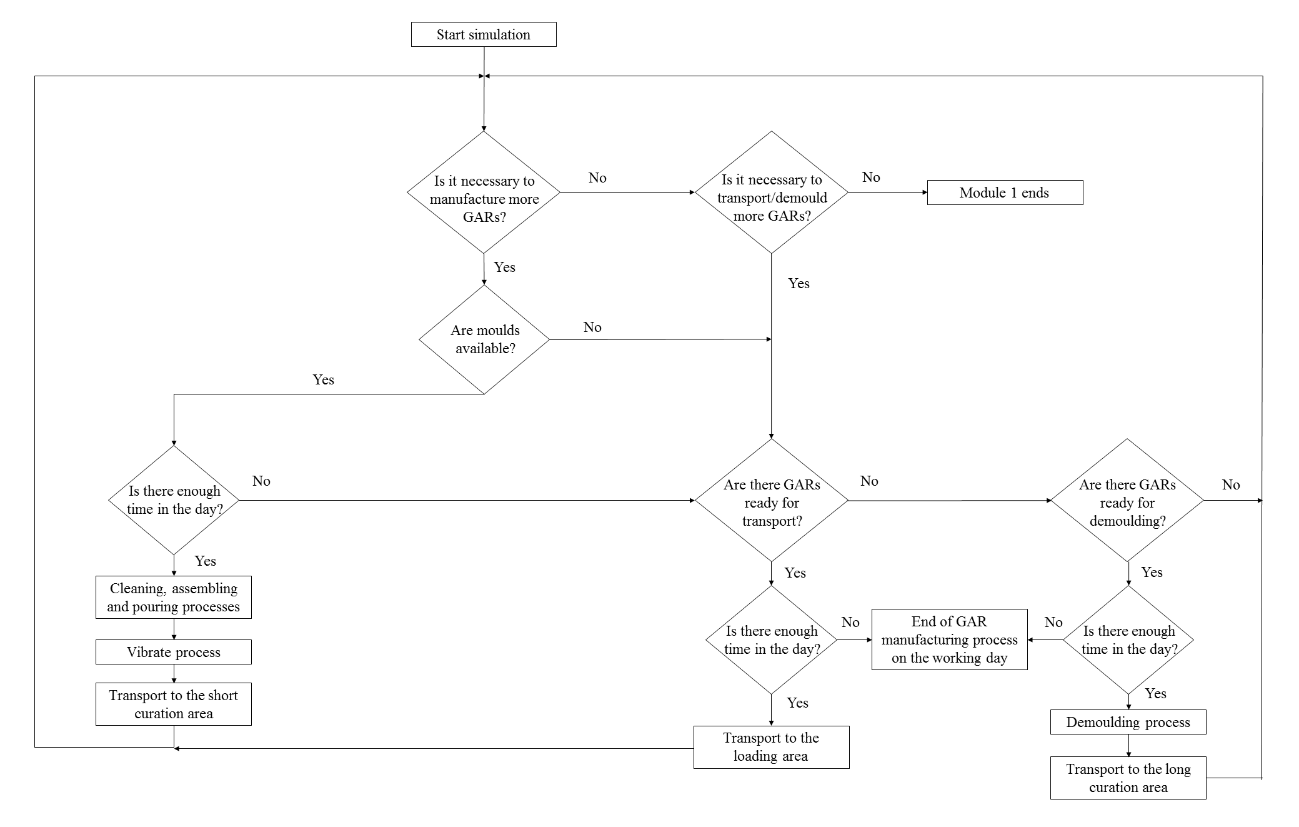


**Figure A.1.** Conceptual flowchart of the manufacturing module (module 1). Source: own elaboration.

As shown in Figure A.1, priority is given to the production of new GARs, as this process is conditioned by the number of moulds. Logically, the number of moulds will always be much lower than the number of GAR units to be produced. In other words, moulds act as the main bottleneck in the manufacturing process. If no moulds are available, priority is given to those artificial reefs that are ready for inland transport, that is, those that have undergone the two curing processes. In this case, a FIFO (Firs In, First Out) approach is adopted. If it is not possible or necessary to manufacture new units and if, in addition, there are no GARs ready for transport, the units that have completed the first curing stage are demoulded. It has been assumed that the manufacturing plant has sufficient space for the storage of materials and, in general, for all the activities that take place there.

The reader should bear in mind that AGARDO takes into account the length of the working day. Therefore, before starting a new activity, AGARDO checks if there is enough time to fully complete it. Nevertheless, these checks could be modified to let certain activities start when they can be completed within a certain percentage. These types of checks are also carried out in modules 2 and 3 (Figures A.2 and A.3, respectively). On the other hand, AGARDO also takes into account that weekends are not working days. The manufacturing module runs continuously until all GARs have been produced.

The second module is the land transport one, Figure A.2. It also runs until all GARs have been transported.


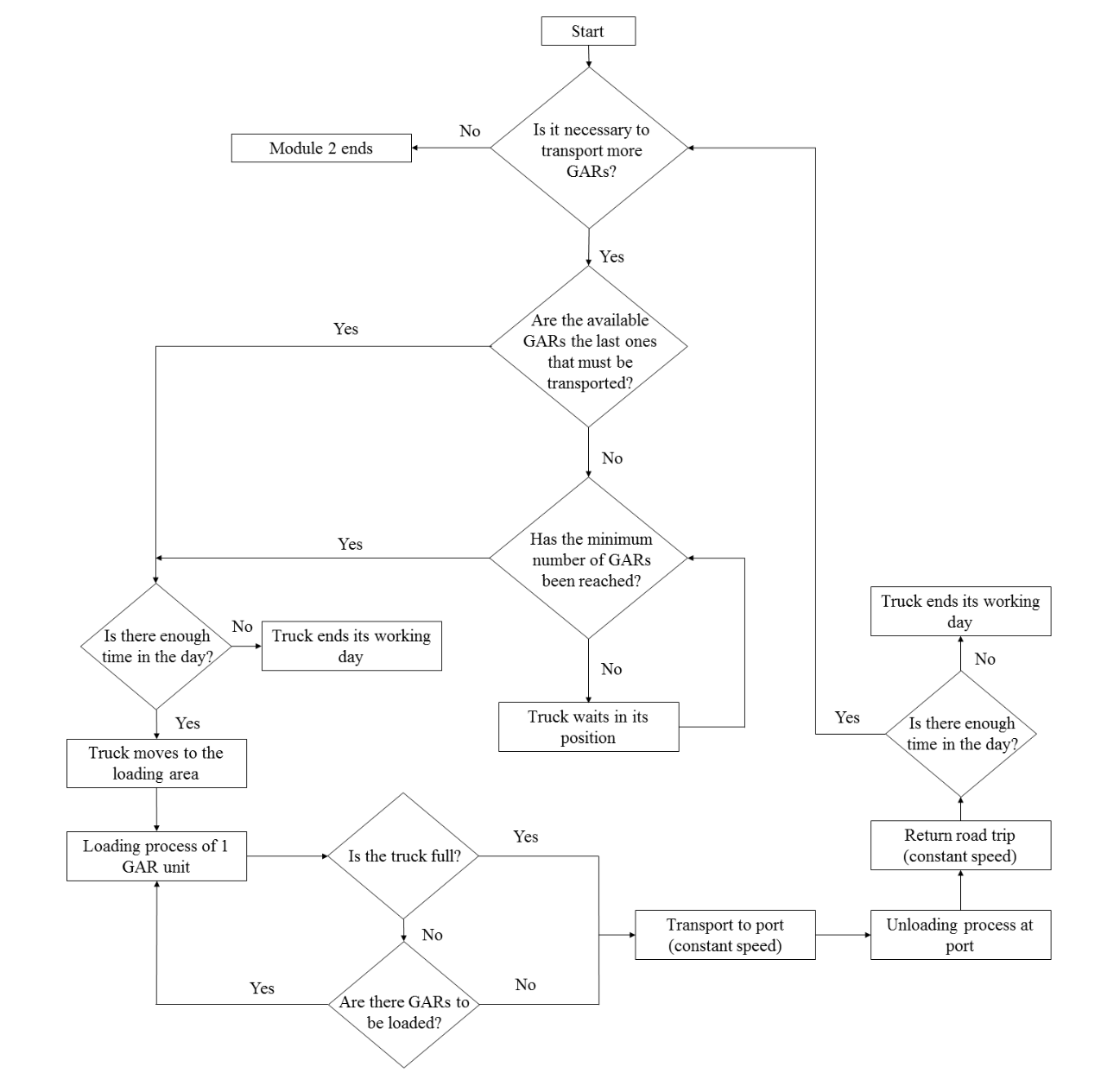


**Figure A.2.** Conceptual flowchart of the ground transport module (module 2). Source: own elaboration*.*

Initially, land transport does not begin until a certain number of reefs have been deposited in the loading area (Figure A.1). This minimum number of GARs depends on the type of vessel to be used for the sea transport. The aim of this is to ensure, as far as possible, extensive and continuous use of the ships, avoiding down times and their corresponding impacts in terms of costs and emissions. This will also serve to guarantee a continuous use of the trucks. In this first version of AGARDO two different types of truck were considered. Nevertheless, a constant speed has been adopted for both options. Similar considerations were defined for the third module, the sea transport one shown in Figure A.3. However, this can be modified in case a more detailed analysis is needed.


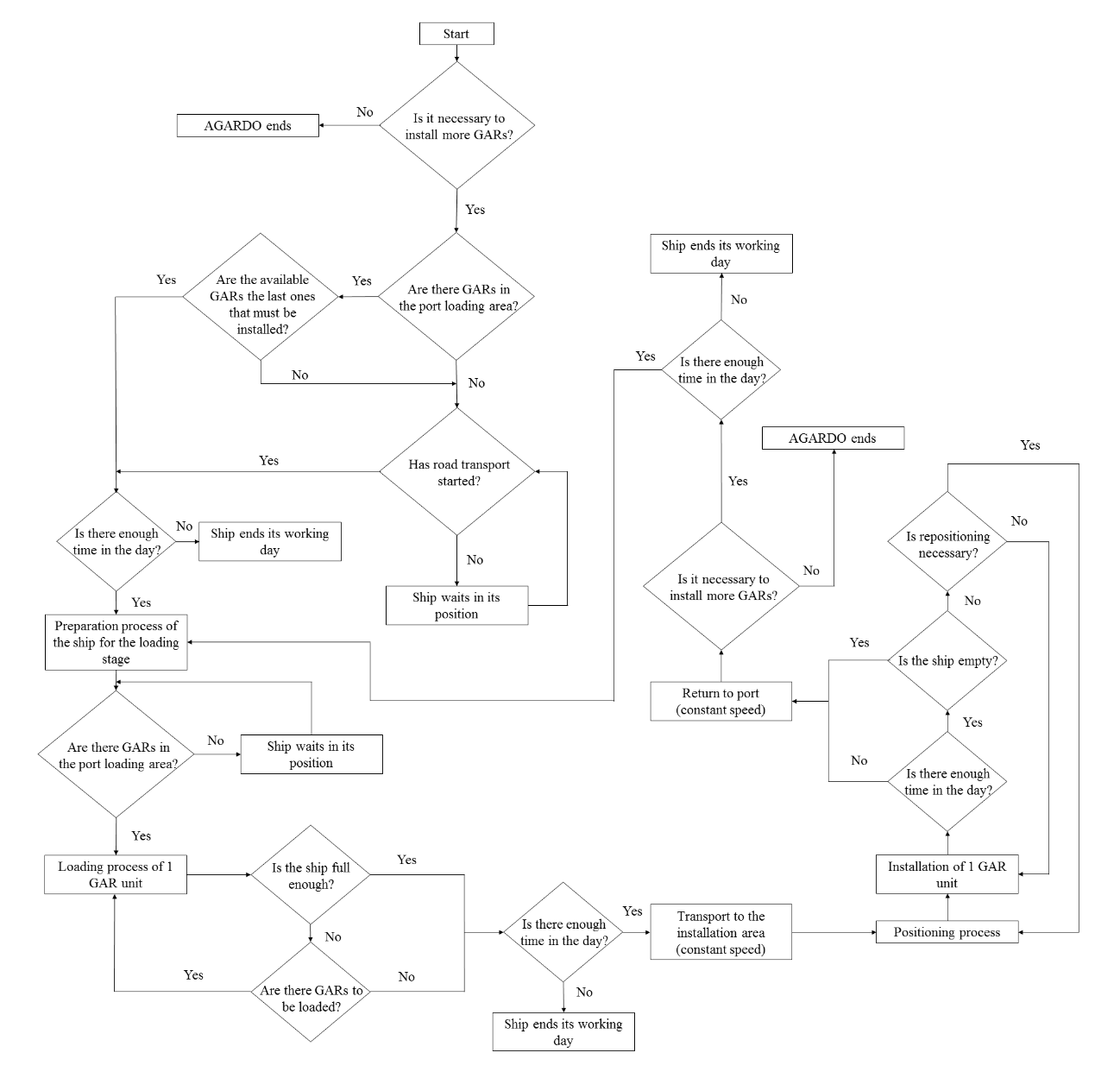


**Figure A.3.** Conceptual flowchart of the sea transport module (module 3). Source: own elaboration.

Module 3 begins when inland transport has started and it runs until the last GAR unit is installed. For the design shown in Figure 1 of the main text, it is very relevant that the different GAR units are positioned relatively accurately as indicated at the beginning of this section. Consequently, the installation process takes longer than in other cases where reefs are thrown overboard.

**A.2. SEA TRANSPORT STAGE: Additional information**


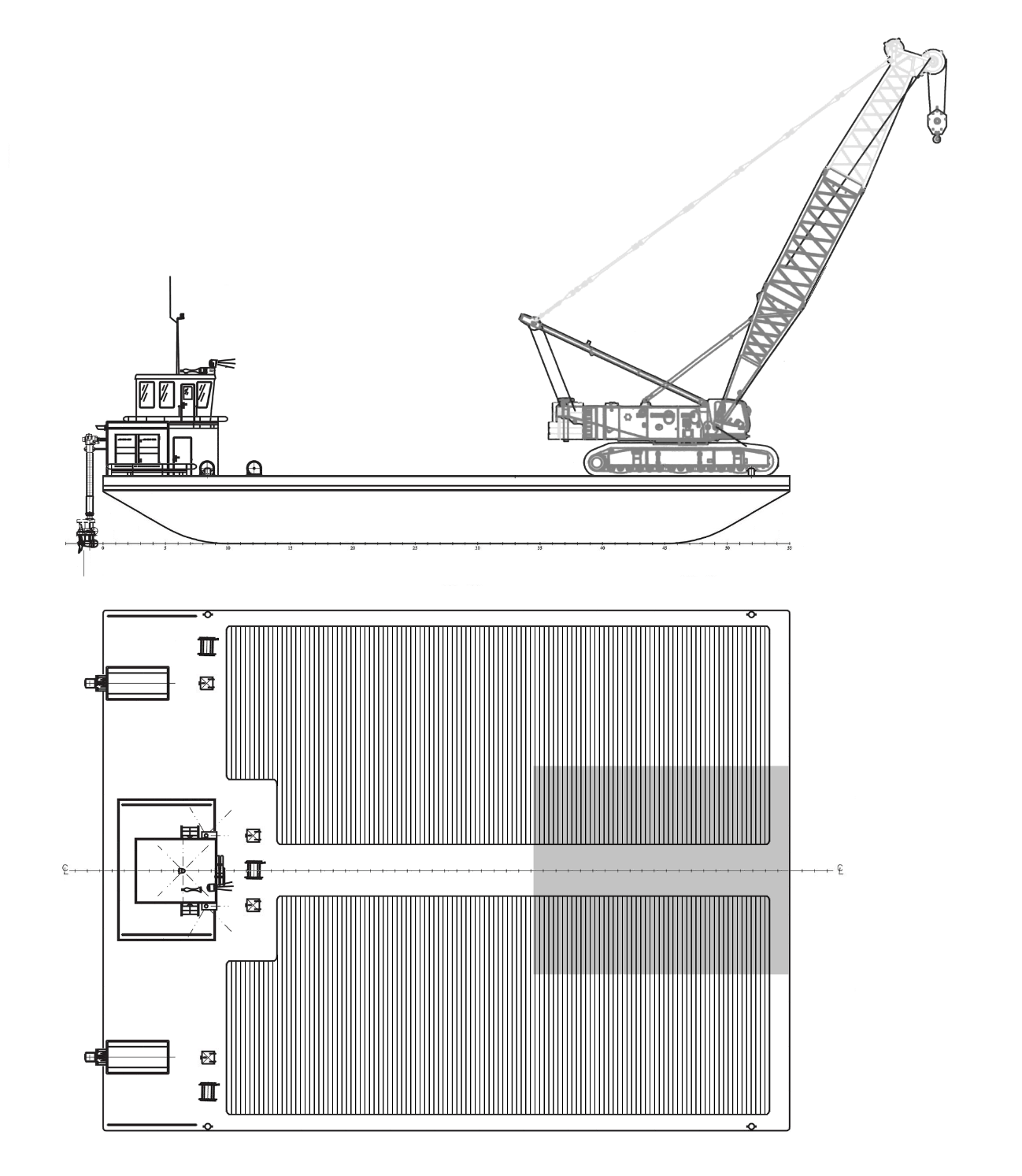


**Figure A.4.** Basic scheme for the conventional barge transporting the GAR units. Source: own elaboration using Maxsurf software, 2024 version (https://www.bentley.com/software/maxsurf/), and AutoCAD software, 2024 version (https://www.autodesk.com/).


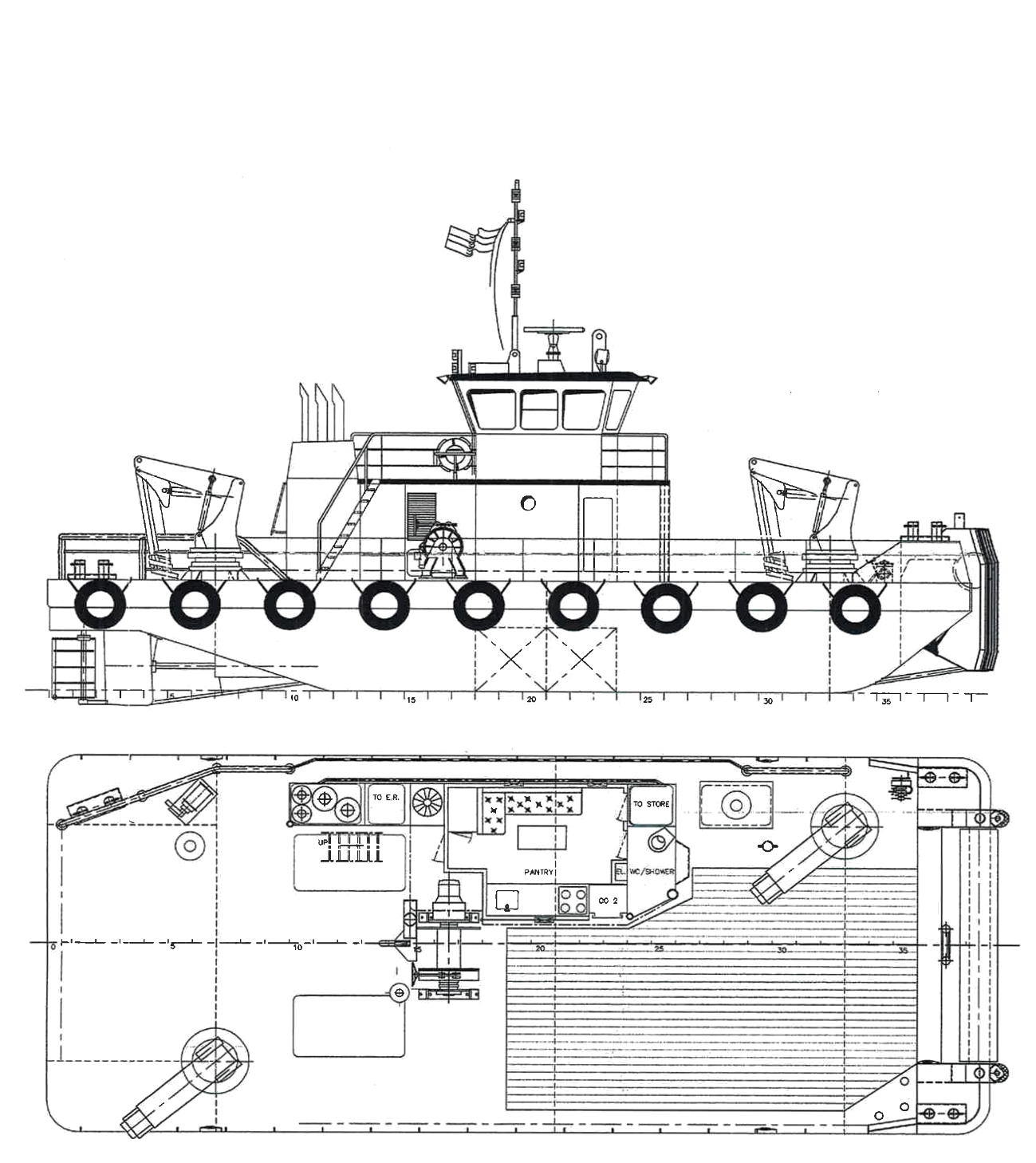


**Figure A.5.** Basic scheme for the LNG-fuelled workboat transporting the GAR units. Source: own elaboration using Maxsurf software, 2024 version (https://www.bentley.com/software/maxsurf/), and AutoCAD software, 2024 version (https://www.autodesk.com/).


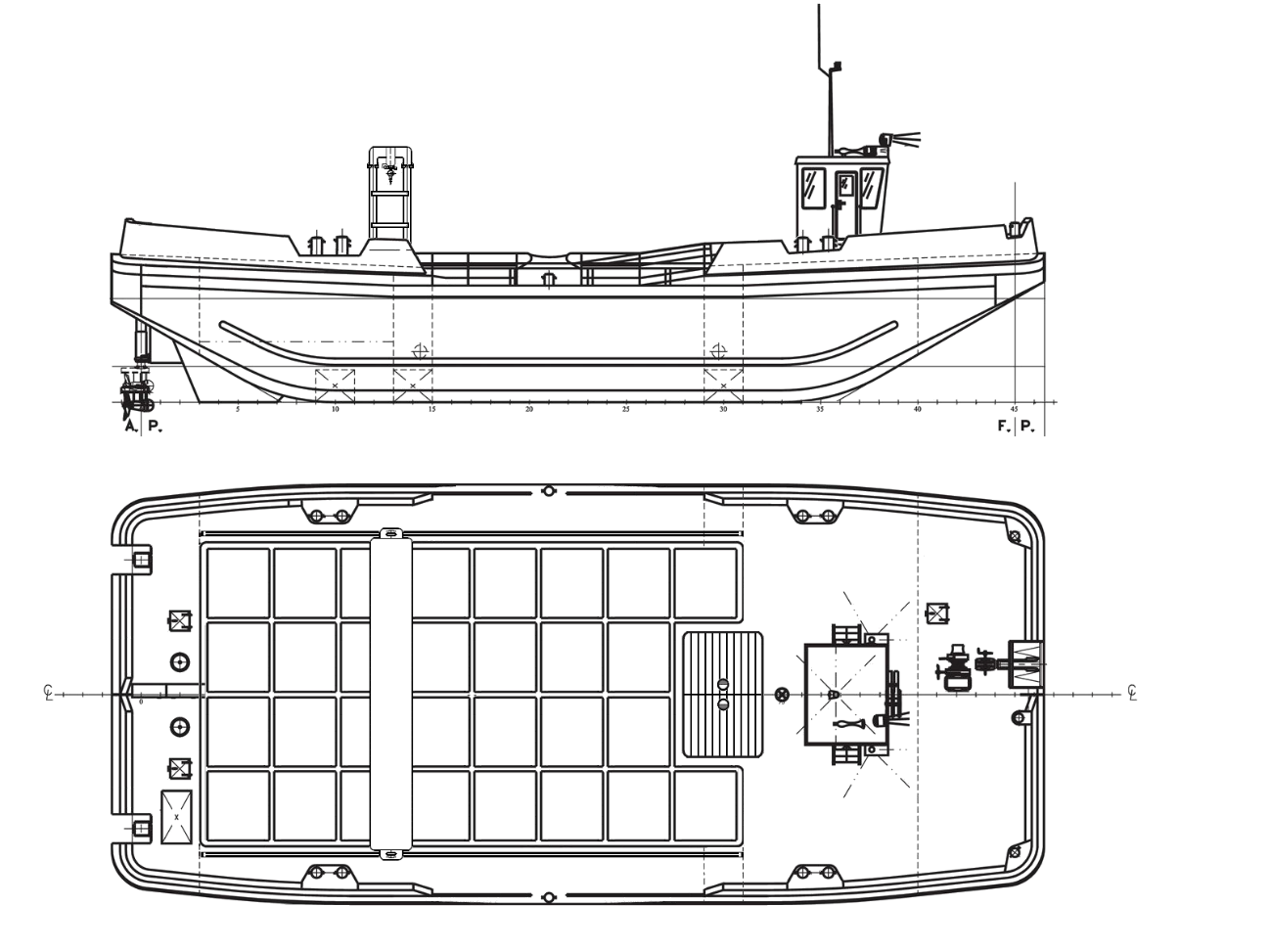


**Figure A.6.** Potential basic design of the electric barge. Source: own elaboration using Maxsurf software, 2024 version (https://www.bentley.com/software/maxsurf/), and AutoCAD software, 2024 version (https://www.autodesk.com/).

**A.3. RESULTS: Additional information**

**Table A.1.** Results for the diesel-fuelled barge with rigid trucks.

| Number of trucks | Number of moulds | Total cost (€) | Total time (days) | Emissions (kg CO_2-eq._) |
| --- | --- | --- | --- | --- |
| 1 | 5 | 446599 | 141.7 | 192568.31 |
| 2 | 5 | 478349 | 141.5 | 140444.66 |
| 3 | 5 | 507599 | 141.5 | 137905.65 |
| 4 | 5 | 527349 | 141.5 | 137905.65 |
| 1 | 7 | 353047 | 105.7 | 195583.39 |
| 2 | 7 | 375297 | 105.7 | 194948.63 |
| 3 | 7 | 397547 | 105.5 | 137588.27 |
| 4 | 7 | 417297 | 105.5 | 137588.27 |
| 1 | 9 | 314736 | 86.5 | 130876.84 |
| 2 | 9 | 328401 | 85.7 | 201184.00 |
| 3 | 9 | 346151 | 85.7 | 189394.55 |
| 4 | 9 | 363901 | 85.5 | 145886.59 |
| 1 | 11 | 288749 | 72.5 | 143618.42 |
| 2 | 11 | 298914 | 71.7 | 211862.63 |
| 3 | 11 | 312414 | 71.7 | 212497.39 |
| 4 | 11 | 325914 | 71.7 | 208371.50 |
| 1 | 13 | 283755 | 65.5 | 133891.91 |
| 2 | 13 | 292170 | 64.5 | 143142.35 |
| 3 | 13 | 303170 | 64.5 | 131194.22 |
| 4 | 13 | 310336 | 63.8 | 232109.08 |
| 1 | 15 | 282846 | 59.5 | 133415.85 |
| 2 | 15 | 289512 | 58.5 | 131352.91 |
| 3 | 15 | 296177 | 57.7 | 210117.07 |
| 4 | 15 | 306927 | 57.7 | 201184.00 |

**Table A.2.** Results for the diesel-fuelled barge with articulated trucks.

| Number of trucks | Number of moulds | Total cost (€) | Total time (days) | Emissions (kg CO_2-eq._) |
| --- | --- | --- | --- | --- |
| 1 | 5 | 465649 | 141.7 | 191140.12 |
| 2 | 5 | 516449 | 141.5 | 136636.15 |
| 3 | 5 | 548049 | 141.5 | 136636.15 |
| 4 | 5 | 579649 | 141.5 | 136636.15 |
| 1 | 7 | 366397 | 105.7 | 186696.86 |
| 2 | 7 | 401997 | 105.5 | 133462.39 |
| 3 | 7 | 433597 | 105.5 | 133462.39 |
| 4 | 7 | 461997 | 105.5 | 133462.39 |
| 1 | 9 | 325536 | 86.5 | 130242.09 |
| 2 | 9 | 349701 | 85.7 | 189235.87 |
| 3 | 9 | 378101 | 85.5 | 144775.77 |
| 4 | 9 | 402501 | 85.5 | 144775.77 |
| 1 | 11 | 293214 | 71.7 | 207101.99 |
| 2 | 11 | 314814 | 71.7 | 206784.62 |
| 3 | 11 | 336414 | 71.7 | 203293.48 |
| 4 | 11 | 356814 | 71.7 | 203293.48 |
| 1 | 13 | 287170 | 64.7 | 207736.75 |
| 2 | 13 | 305970 | 64.5 | 136794.83 |
| 3 | 13 | 319736 | 63.8 | 227983.19 |
| 4 | 13 | 337336 | 63.8 | 224333.37 |
| 1 | 15 | 285362 | 58.7 | 207260.68 |
| 2 | 15 | 298327 | 57.8 | 215717.68 |
| 3 | 15 | 315527 | 57.7 | 196740.74 |
| 4 | 15 | 332727 | 57.6 | 162959.27 |

**Table A.3.** Results for the LNG-fuelled workboat with rigid trucks.

| Number of trucks | Number of moulds | Total cost (€) | Total time (days) | Emissions (kg CO_2-eq._) |
| --- | --- | --- | --- | --- |
| 1 | 5 | 536573 | 134.5 | 205022.99 |
| 2 | 5 | 568323 | 134.5 | 203277.42 |
| 3 | 5 | 598573 | 134.5 | 201055.79 |
| 4 | 5 | 618323 | 134.5 | 201055.79 |
| 1 | 7 | 412121 | 98.7 | 303790.05 |
| 2 | 7 | 434353 | 98.7 | 169261.76 |
| 3 | 7 | 456603 | 98.5 | 132948.16 |
| 4 | 7 | 476353 | 98.5 | 132948.16 |
| 1 | 9 | 358354 | 79.4 | 144978.27 |
| 2 | 9 | 370821 | 78.8 | 195260.65 |
| 3 | 9 | 388571 | 78.7 | 173326.11 |
| 4 | 9 | 406321 | 78.7 | 161009.99 |
| 1 | 11 | 320476 | 65.6 | 148279.35 |
| 2 | 11 | 334481 | 65.4 | 145771.71 |
| 3 | 11 | 342955 | 64.8 | 225734.18 |
| 4 | 11 | 356449 | 64.8 | 182468.47 |
| 1 | 13 | 309551 | 58.6 | 232000.38 |
| 2 | 13 | 316775 | 57.6 | 218908.40 |
| 3 | 13 | 327757 | 57.7 | 133491.60 |
| 4 | 13 | 341019 | 57.5 | 156809.54 |
| 1 | 15 | 309162 | 53.6 | 192779.39 |
| 2 | 15 | 309609 | 51.7 | 202071.65 |
| 3 | 15 | 314838 | 50.7 | 253582.38 |
| 4 | 15 | 331109 | 51.4 | 144502.21 |

**Table A.4.** Results for the LNG-fuelled workboat with articulated trucks.

| Number of trucks | Number of moulds | Total cost (€) | Total time (days) | Emissions (kg CO_2-eq._) |
| --- | --- | --- | --- | --- |
| 1 | 5 | 555611 | 134.6 | 147168.53 |
| 2 | 5 | 606411 | 134.6 | 151867.63 |
| 3 | 5 | 638011 | 134.6 | 151867.63 |
| 4 | 5 | 670811 | 134.6 | 151867.63 |
| 1 | 7 | 425465 | 98.5 | 187963.81 |
| 2 | 7 | 461053 | 98.5 | 128822.27 |
| 3 | 7 | 492653 | 98.5 | 128822.27 |
| 4 | 7 | 525453 | 98.5 | 128822.27 |
| 1 | 9 | 369148 | 79.4 | 115932.94 |
| 2 | 9 | 392121 | 78.7 | 164342.44 |
| 3 | 9 | 420521 | 78.7 | 159899.17 |
| 4 | 9 | 446521 | 78.7 | 159899.17 |
| 1 | 11 | 328787 | 65.4 | 175030.52 |
| 2 | 11 | 345361 | 64.8 | 276909.95 |
| 3 | 11 | 366949 | 64.8 | 187961.00 |
| 4 | 11 | 387349 | 64.8 | 187961.00 |
| 1 | 13 | 311769 | 57.8 | 234232.99 |
| 2 | 13 | 330563 | 57.6 | 152026.32 |
| 3 | 13 | 348169 | 57.4 | 141645.82 |
| 4 | 13 | 365769 | 57.4 | 138630.75 |
| 1 | 15 | 305209 | 51.8 | 232646.11 |
| 2 | 15 | 316988 | 50.7 | 249932.56 |
| 3 | 15 | 339609 | 51.4 | 140693.70 |
| 4 | 15 | 351377 | 50.8 | 187802.32 |

**Table A.5.** Results for the electric barge with rigid trucks.

| Number of trucks | Number of moulds | Total cost (€) | Total time (days) | Emissions (kg CO_2-eq._) |
| --- | --- | --- | --- | --- |
| 1 | 5 | 271179 | 133.7 | 40684.52 |
| 2 | 5 | 302923 | 133.6 | 34889.72 |
| 3 | 5 | 332173 | 133.6 | 32350.72 |
| 4 | 5 | 351923 | 133.6 | 32350.72 |
| 1 | 7 | 221067 | 95.9 | 42586.07 |
| 2 | 7 | 243311 | 95.8 | 38063.48 |
| 3 | 7 | 265561 | 95.6 | 32033.34 |
| 4 | 7 | 284311 | 95.6 | 32033.34 |
| 1 | 9 | 204556 | 78.6 | 35365.79 |
| 2 | 9 | 219896 | 77.8 | 34635.45 |
| 3 | 9 | 237646 | 77.7 | 36555.92 |
| 4 | 9 | 255396 | 77.7 | 33540.85 |
| 1 | 11 | 195298 | 64.7 | 37904.80 |
| 2 | 11 | 207132 | 63.9 | 34889.72 |
| 3 | 11 | 222804 | 64.4 | 29215.73 |
| 4 | 11 | 236304 | 64.4 | 26203.36 |
| 1 | 13 | 201084 | 58.6 | 38539.55 |
| 2 | 13 | 208759 | 56.6 | 34947.42 |
| 3 | 13 | 219753 | 56.6 | 35048.41 |
| 4 | 13 | 230765 | 56.4 | 30819.21 |
| 1 | 15 | 204455 | 51.6 | 37904.80 |
| 2 | 15 | 212790 | 50.6 | 36159.23 |
| 3 | 15 | 223796 | 50.6 | 33683.32 |
| 4 | 15 | 234296 | 50.5 | 32731.19 |

**Table A.6.** Results for the electric barge with articulated trucks.

| Number of trucks | Number of moulds | Total cost (€) | Total time (days) | Emissions (kg CO_2-eq._) |
| --- | --- | --- | --- | --- |
| 1 | 5 | 290229 | 133.7 | 38618.87 |
| 2 | 5 | 341029 | 133.7 | 34334.29 |
| 3 | 5 | 372629 | 133.7 | 34334.29 |
| 4 | 5 | 404229 | 133.7 | 34334.29 |
| 1 | 7 | 234417 | 95.7 | 28758.59 |
| 2 | 7 | 270011 | 95.6 | 27907.45 |
| 3 | 7 | 301211 | 95.6 | 27907.45 |
| 4 | 7 | 331211 | 95.6 | 27907.45 |
| 1 | 9 | 215356 | 78.6 | 33937.60 |
| 2 | 9 | 241196 | 77.7 | 36717.32 |
| 3 | 9 | 269596 | 77.7 | 33067.49 |
| 4 | 9 | 295596 | 77.7 | 33067.49 |
| 1 | 11 | 201432 | 63.9 | 31398.59 |
| 2 | 11 | 225204 | 64.4 | 24775.17 |
| 3 | 11 | 246804 | 64.4 | 22397.55 |
| 4 | 11 | 267204 | 64.4 | 22397.55 |
| 1 | 13 | 205925 | 57.4 | 25883.28 |
| 2 | 13 | 223759 | 56.6 | 28911.87 |
| 3 | 13 | 240165 | 56.4 | 28911.48 |
| 4 | 13 | 257765 | 56.4 | 26213.78 |
| 1 | 15 | 210811 | 51.4 | 25885.99 |
| 2 | 15 | 225446 | 50.4 | 24616.48 |
| 3 | 15 | 240480 | 49.9 | 39598.03 |
| 4 | 15 | 257680 | 49.9 | 36900.33 |

**Table A.7.** Detailed results for costs and emissions. Diesel-fuelled barge with rigid trucks. Cost of moulds not included.

| Number of trucks | Number of moulds | Cost of trucks (€) | Cost of the ship (€) | Truck emissions (kg CO_2-eq._) | Ship emissions (kg CO_2-eq._) |
| --- | --- | --- | --- | --- | --- |
| 1 | 5 | 31750 | 374849 | 17138.30 | 175430.01 |
| 2 | 5 | 63500 | 374849 | 15234.05 | 125210.61 |
| 3 | 5 | 92750 | 374849 | 12695.04 | 125210.61 |
| 4 | 5 | 112500 | 374849 | 12695.04 | 125210.61 |
| 1 | 7 | 22250 | 274797 | 20153.38 | 175430.01 |
| 2 | 7 | 44500 | 274797 | 19518.62 | 175430.01 |
| 3 | 7 | 66750 | 274797 | 12377.66 | 125210.61 |
| 4 | 7 | 86500 | 274797 | 12377.66 | 125210.61 |
| 1 | 9 | 18000 | 224736 | 15710.11 | 115166.73 |
| 2 | 9 | 35500 | 220901 | 15710.11 | 185473.89 |
| 3 | 9 | 53250 | 220901 | 13964.54 | 175430.01 |
| 4 | 9 | 71000 | 220901 | 10632.10 | 135254.49 |
| 1 | 11 | 14500 | 186249 | 18407.81 | 125210.61 |
| 2 | 11 | 28500 | 182414 | 16344.86 | 195517.77 |
| 3 | 11 | 42000 | 182414 | 16979.62 | 195517.77 |
| 4 | 11 | 55500 | 182414 | 12853.73 | 195517.77 |
| 1 | 13 | 12750 | 167005 | 18725.18 | 115166.73 |
| 2 | 13 | 25000 | 163170 | 17931.74 | 125210.61 |
| 3 | 13 | 36000 | 163170 | 16027.49 | 115166.73 |
| 4 | 13 | 47000 | 159336 | 16503.55 | 215605.53 |
| 1 | 15 | 11250 | 151596 | 18249.12 | 115166.73 |
| 2 | 15 | 21750 | 147762 | 16186.18 | 115166.73 |
| 3 | 15 | 32250 | 143927 | 14599.30 | 195517.77 |
| 4 | 15 | 43000 | 143927 | 15710.11 | 185473.89 |

**Table A.8.** Detailed results for costs and emissions. Diesel-fuelled barge with articulated trucks. Cost of moulds not included.

| Number of trucks | Number of moulds | Cost of trucks (€) | Cost of the ship (€) | Truck emissions (kg CO_2-eq._) | Ship emissions (kg CO_2-eq._) |
| --- | --- | --- | --- | --- | --- |
| 1 | 5 | 50800 | 374849 | 15710.11 | 175430.01 |
| 2 | 5 | 101600 | 374849 | 11425.54 | 125210.61 |
| 3 | 5 | 133200 | 374849 | 11425.54 | 125210.61 |
| 4 | 5 | 164800 | 374849 | 11425.54 | 125210.61 |
| 1 | 7 | 35600 | 274797 | 11266.85 | 175430.01 |
| 2 | 7 | 71200 | 274797 | 8251.78 | 125210.61 |
| 3 | 7 | 102800 | 274797 | 8251.78 | 125210.61 |
| 4 | 7 | 131200 | 274797 | 8251.78 | 125210.61 |
| 1 | 9 | 28800 | 224736 | 15075.36 | 115166.73 |
| 2 | 9 | 56800 | 220901 | 13805.86 | 175430.01 |
| 3 | 9 | 85200 | 220901 | 9521.28 | 135254.49 |
| 4 | 9 | 109600 | 220901 | 9521.28 | 135254.49 |
| 1 | 11 | 22800 | 182414 | 11584.22 | 195517.77 |
| 2 | 11 | 44400 | 182414 | 11266.85 | 195517.77 |
| 3 | 11 | 66000 | 182414 | 7775.71 | 195517.77 |
| 4 | 11 | 86400 | 182414 | 7775.71 | 195517.77 |
| 1 | 13 | 20000 | 163170 | 12218.98 | 195517.77 |
| 2 | 13 | 38800 | 163170 | 11584.22 | 125210.61 |
| 3 | 13 | 56400 | 159336 | 12377.66 | 215605.53 |
| 4 | 13 | 74000 | 159336 | 8727.84 | 215605.53 |
| 1 | 15 | 17600 | 147762 | 11742.91 | 195517.77 |
| 2 | 15 | 34400 | 143927 | 10156.03 | 205561.65 |
| 3 | 15 | 51600 | 143927 | 11266.85 | 185473.89 |
| 4 | 15 | 68800 | 143927 | 7617.02 | 155342.25 |

**Table A.9.** Detailed results for costs and emissions. LNG-fuelled workboat with rigid trucks. Cost of moulds not included.

| Number of trucks | Number of moulds | Cost of trucks (€) | Cost of the ship (€) | Truck emissions (kg CO_2-eq._) | Ship emissions (kg CO_2-eq._) |
| --- | --- | --- | --- | --- | --- |
| 1 | 5 | 31750 | 464823 | 16662.24 | 188360.75 |
| 2 | 5 | 63500 | 464823 | 14916.67 | 188360.75 |
| 3 | 5 | 93750 | 464823 | 12695.04 | 188360.75 |
| 4 | 5 | 113500 | 464823 | 12695.04 | 188360.75 |
| 1 | 7 | 22250 | 333871 | 19201.25 | 284588.80 |
| 2 | 7 | 44500 | 333853 | 18883.87 | 150377.89 |
| 3 | 7 | 66750 | 333853 | 12377.66 | 120570.49 |
| 4 | 7 | 86500 | 333853 | 12377.66 | 120570.49 |
| 1 | 9 | 18000 | 268354 | 15075.36 | 129902.91 |
| 2 | 9 | 35500 | 263321 | 15075.36 | 180185.29 |
| 3 | 9 | 53250 | 263321 | 13012.42 | 160313.69 |
| 4 | 9 | 71000 | 263321 | 10632.10 | 150377.89 |
| 1 | 11 | 14500 | 217976 | 17773.06 | 130506.29 |
| 2 | 11 | 28500 | 217981 | 15868.80 | 129902.91 |
| 3 | 11 | 42000 | 212955 | 16344.86 | 209389.31 |
| 4 | 11 | 55500 | 212949 | 12218.98 | 170249.49 |
| 1 | 13 | 12750 | 192801 | 18090.43 | 213909.95 |
| 2 | 13 | 25000 | 187775 | 17773.06 | 201135.35 |
| 3 | 13 | 36000 | 187757 | 15710.11 | 117781.49 |
| 4 | 13 | 49250 | 187769 | 15551.42 | 141258.11 |
| 1 | 15 | 11500 | 177662 | 17455.68 | 175323.71 |
| 2 | 15 | 22000 | 167609 | 15392.74 | 186678.91 |
| 3 | 15 | 32250 | 162588 | 14123.23 | 239459.15 |
| 4 | 15 | 43500 | 167609 | 14599.30 | 129902.91 |

**Table A.10.** Detailed results for costs and emissions. LNG-fuelled workboat with articulated trucks. Cost of moulds not included.

| Number of trucks | Number of moulds | Cost of trucks (€) | Cost of the ship (€) | Truck emissions (kg CO_2-eq._) | Ship emissions (kg CO_2-eq._) |
| --- | --- | --- | --- | --- | --- |
| 1 | 5 | 50800 | 464811 | 16662.24 | 130506.29 |
| 2 | 5 | 101600 | 464811 | 11425.54 | 140442.09 |
| 3 | 5 | 133200 | 464811 | 11425.54 | 140442.09 |
| 4 | 5 | 166000 | 464811 | 11425.54 | 140442.09 |
| 1 | 7 | 35600 | 333865 | 12377.66 | 175586.15 |
| 2 | 7 | 71200 | 333853 | 8251.78 | 120570.49 |
| 3 | 7 | 102800 | 333853 | 8251.78 | 120570.49 |
| 4 | 7 | 135600 | 333853 | 8251.78 | 120570.49 |
| 1 | 9 | 28800 | 268348 | 15234.05 | 100698.89 |
| 2 | 9 | 56800 | 263321 | 13964.54 | 150377.89 |
| 3 | 9 | 85200 | 263321 | 9521.28 | 150377.89 |
| 4 | 9 | 111200 | 263321 | 9521.28 | 150377.89 |
| 1 | 11 | 22800 | 217987 | 12218.98 | 162811.55 |
| 2 | 11 | 44400 | 212961 | 11901.60 | 265008.35 |
| 3 | 11 | 66000 | 212949 | 7775.71 | 180185.29 |
| 4 | 11 | 86400 | 212949 | 7775.71 | 180185.29 |
| 1 | 13 | 20000 | 187769 | 13488.48 | 220744.51 |
| 2 | 13 | 38800 | 187763 | 11584.22 | 140442.09 |
| 3 | 13 | 56400 | 187769 | 11742.91 | 129902.91 |
| 4 | 13 | 74000 | 187769 | 8727.84 | 129902.91 |
| 1 | 15 | 17600 | 167609 | 11901.60 | 220744.51 |
| 2 | 15 | 34400 | 162588 | 10473.41 | 239459.15 |
| 3 | 15 | 52000 | 167609 | 10790.78 | 129902.91 |
| 4 | 15 | 68800 | 162577 | 7617.02 | 180185.29 |

**Table A.11.** Detailed results for costs and emissions. Electric barge with rigid trucks. Cost of moulds not included.

| Number of trucks | Number of moulds | Cost of trucks (€) | Cost of the ship (€) | Truck emissions (kg CO_2-eq._) | Ship emissions (kg CO_2-eq._) |
| --- | --- | --- | --- | --- | --- |
| 1 | 5 | 31750 | 199429 | 17138.30 | 23546.21 |
| 2 | 5 | 63500 | 199423 | 15234.05 | 19655.68 |
| 3 | 5 | 92750 | 199423 | 12695.04 | 19655.68 |
| 4 | 5 | 112500 | 199423 | 12695.04 | 19655.68 |
| 1 | 7 | 22250 | 142817 | 19677.31 | 22908.76 |
| 2 | 7 | 44500 | 142811 | 18407.81 | 19655.68 |
| 3 | 7 | 66750 | 142811 | 12377.66 | 19655.68 |
| 4 | 7 | 85500 | 142811 | 12377.66 | 19655.68 |
| 1 | 9 | 18000 | 114556 | 15710.11 | 19655.68 |
| 2 | 9 | 35500 | 112396 | 15551.42 | 19084.03 |
| 3 | 9 | 53250 | 112396 | 13647.17 | 22908.76 |
| 4 | 9 | 71000 | 112396 | 10632.10 | 22908.76 |
| 1 | 11 | 14500 | 92798 | 18249.12 | 19655.68 |
| 2 | 11 | 28500 | 90632 | 15234.05 | 19655.68 |
| 3 | 11 | 42000 | 92804 | 15868.80 | 13346.93 |
| 4 | 11 | 55500 | 92804 | 12218.98 | 13984.39 |
| 1 | 13 | 13000 | 84084 | 18883.87 | 19655.68 |
| 2 | 13 | 25000 | 79759 | 17138.30 | 17809.12 |
| 3 | 13 | 36000 | 79753 | 15392.74 | 19655.68 |
| 4 | 13 | 47000 | 79765 | 15392.74 | 15426.47 |
| 1 | 15 | 11250 | 73205 | 18249.12 | 19655.68 |
| 2 | 15 | 21750 | 71040 | 16503.55 | 19655.68 |
| 3 | 15 | 32750 | 71046 | 14599.30 | 19084.03 |
| 4 | 15 | 43250 | 71046 | 13647.17 | 19084.03 |

**Table A.12.** Detailed results for costs and emissions. Electric barge with articulated trucks. Cost of moulds not included.

| Number of trucks | Number of moulds | Cost of trucks (€) | Cost of the ship (€) | Truck emissions (kg CO_2-eq._) | Ship emissions (kg CO_2-eq._) |
| --- | --- | --- | --- | --- | --- |
| 1 | 5 | 50800 | 199429 | 15710.11 | 22908.76 |
| 2 | 5 | 101600 | 199429 | 11425.54 | 22908.76 |
| 3 | 5 | 133200 | 199429 | 11425.54 | 22908.76 |
| 4 | 5 | 164800 | 199429 | 11425.54 | 22908.76 |
| 1 | 7 | 35600 | 142817 | 10949.47 | 17809.12 |
| 2 | 7 | 71200 | 142811 | 8251.78 | 19655.68 |
| 3 | 7 | 102400 | 142811 | 8251.78 | 19655.68 |
| 4 | 7 | 132400 | 142811 | 8251.78 | 19655.68 |
| 1 | 9 | 28800 | 114556 | 14281.92 | 19655.68 |
| 2 | 9 | 56800 | 112396 | 13171.10 | 23546.21 |
| 3 | 9 | 85200 | 112396 | 9521.28 | 23546.21 |
| 4 | 9 | 111200 | 112396 | 9521.28 | 23546.21 |
| 1 | 11 | 22800 | 90632 | 11742.91 | 19655.68 |
| 2 | 11 | 44400 | 92804 | 10790.78 | 13984.39 |
| 3 | 11 | 66000 | 92804 | 7775.71 | 14621.84 |
| 4 | 11 | 86400 | 92804 | 7775.71 | 14621.84 |
| 1 | 13 | 20000 | 81925 | 12536.35 | 13346.93 |
| 2 | 13 | 40000 | 79759 | 12377.66 | 16534.21 |
| 3 | 13 | 56400 | 79765 | 11425.54 | 17485.94 |
| 4 | 13 | 74000 | 79765 | 8727.84 | 17485.94 |
| 1 | 15 | 17600 | 73211 | 11901.60 | 13984.39 |
| 2 | 15 | 34400 | 71046 | 10632.10 | 13984.39 |
| 3 | 15 | 51600 | 68880 | 10314.72 | 29283.31 |
| 4 | 15 | 68800 | 68880 | 7617.02 | 29283.31 |


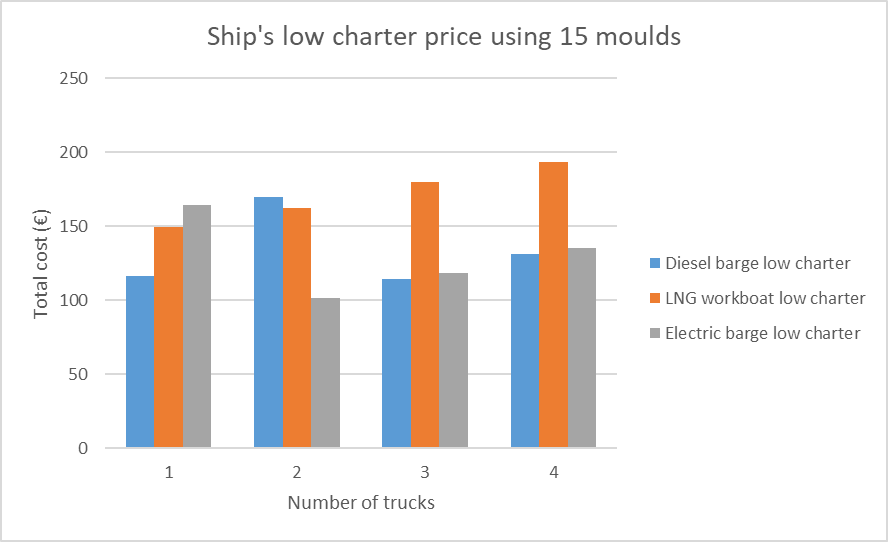


**Figure A.7.** Results of the sensitivity analysis for a low charter price. Source: own elaboration.


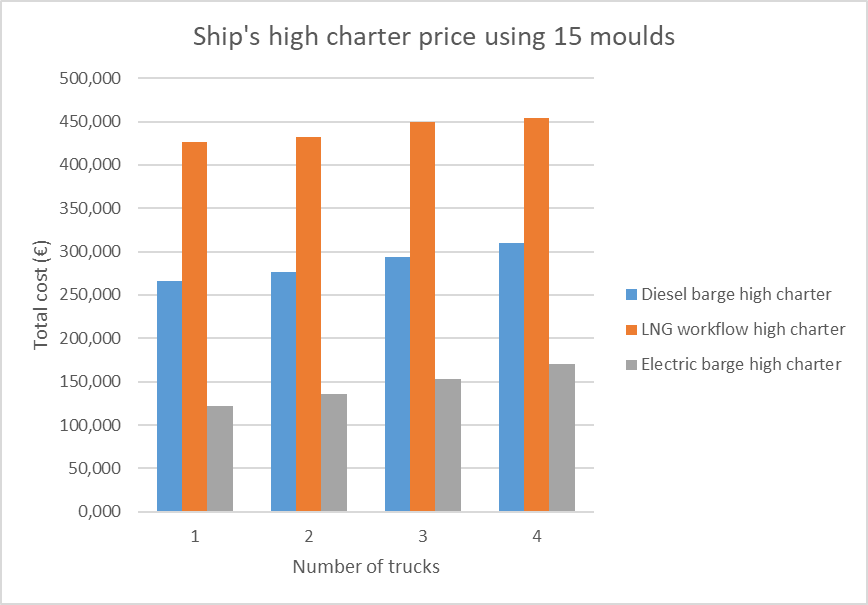


**Figure A.8.** Results of the sensitivity analysis for a high charter price. Source: own elaboration.
